# Supplementary material for: The autophagy receptor p62/SQST-1 promotes proteostasis and longevity in C. elegans by inducing autophagy
Source: Nat Commun. 2019 Dec 11;10:5648. doi: 10.1038/s41467-019-13540-4 (PMC6906454; doi:10.1038/s41467-019-13540-4)
Supplement: Supplementary file 1 — Supplementary Information [file 41467_2019_13540_MOESM1_ESM.pdf]

SUPPLEMENTARY INFORMATION

The autophagy receptor p62/SQST-1 promotes proteostasis and longevity in *C. elegans* by inducing autophagy

Kumsta et. al.

Contents:

- Supplementary Figure 1-12
- Supplementary Tables 1-6
- Supplementary Methods
- Supplementary References

1. Supplementary Figures and Legends

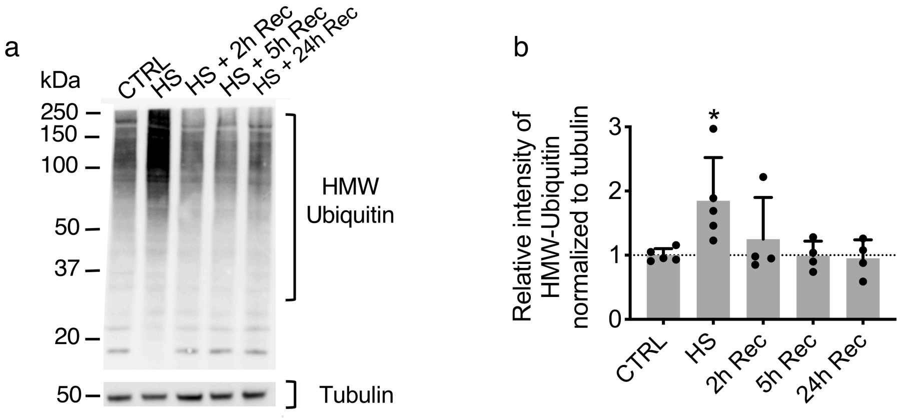

Supplementary Figure 1: Heat shock leads to reversible protein ubiquitination

(a) Representative Western blot of wild-type (WT, N2) animals (N=50 animals per sample) either kept at 20°C for control conditions (CTRL) or subjected to a hormetic heat shock for 1 h at 36 °C on day 1 of adulthood (HS) followed by 0, 2, 5, and 24 h of recovery (Rec) using antibodies against ubiquitin, indicating high molecular weight (HMW) ubiquitin-conjugates and tubulin.

(b) Quantification of the abundance of HMW-ubiquitin normalized to tubulin and relative to control conditions (CTRL) of four independent experiments. Error bars indicate SD. *$P<0.05$, by one-way ANOVA with Dunnett's multiple comparisons test.

Source data are provided in the Source Data file.

Supplementary Figure 2

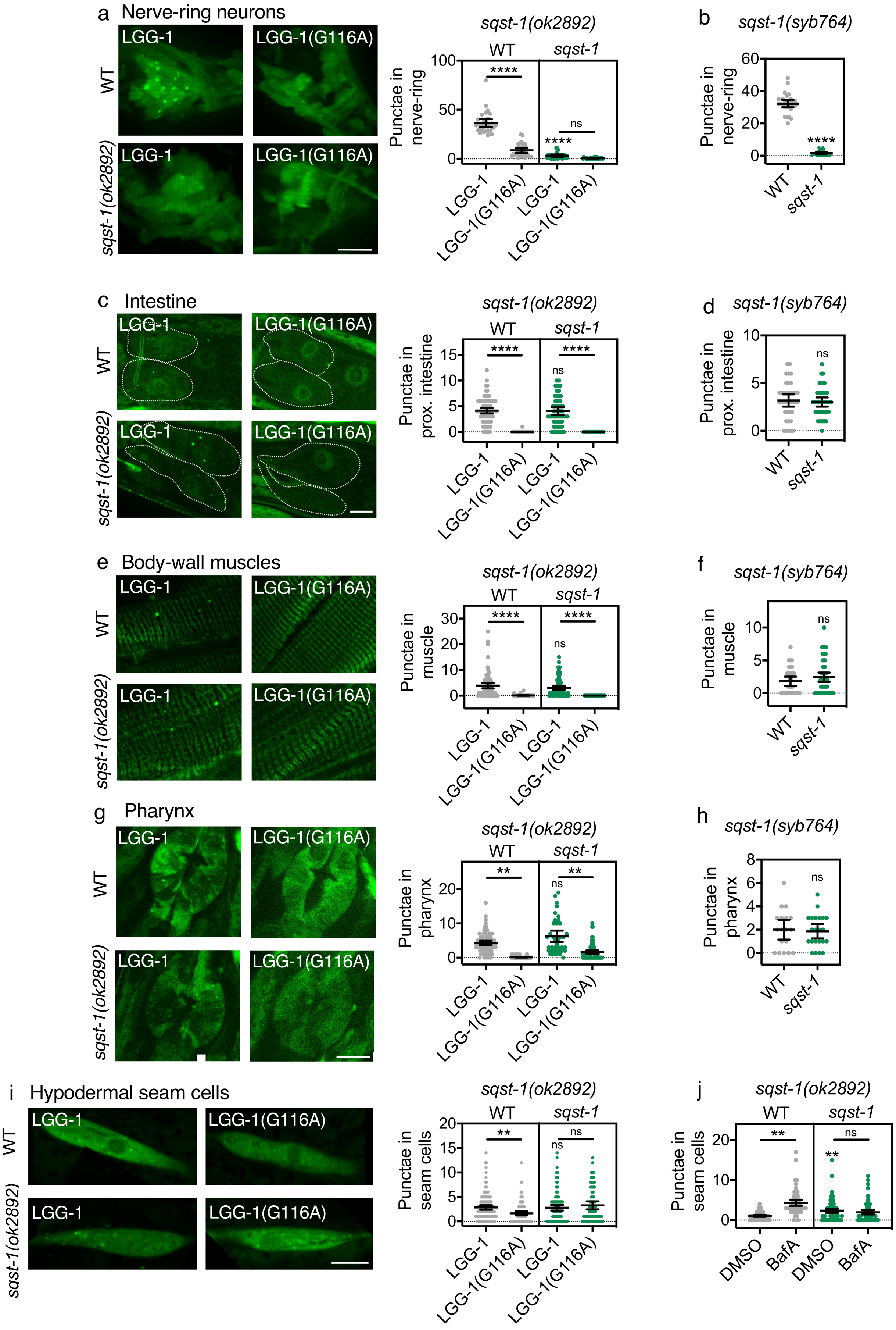

Supplementary Figure 2: Reduced function of *sqst-1* decreases autophagosome formation specifically in nerve-ring neurons of *C. elegans*.

(a) GFP::LGG-1/Atg8-positive punctae were quantified from three independent experiments in nerve-ring neurons of wild-type (WT) and *sqst-1(ok2892)* animals expressing *rgef-1p::gfp::lgg-1* or *rgef-1p::gfp::lgg-1(G116A).* WT-LGG-1, N=31; WT-LGG-1(G116A), N=30; *sqst-1*-LGG-1, N=27, *sqst-1*-LGG-1(G116A), N=27 animals. Error bars indicate 95% CI. ns: P>0.5, ****P<0.0001, by two-way ANOVA with Tukey's multiple comparisons test. Scale bar: 10 µm.

GFP-tagged LGG-1(G116A) mutant protein is defective in lipidation and therefore autophagosome targeting[1], rendering it diffuse in the cytosol, and is used to verify that GFP::LGG-1/Atg8-positive punctae are representing autophagic vesicles. Unexpectedly, nerve-ring neurons expressing GFP-tagged LGG-1(G116A) showed a propensity to form some LGG-1/Atg8-lipidation independent structures. This may be due to the increased expression of LGG-1/Atg8 under the control of the pan-neuronal *rgef-1* promoter, similar to the increased formation of LC3(G120A) punctae observed in cells transfected to overexpressing LC3[2]. The nature of these lipidation-independent GFP::LGG-1/Atg8-positive structures is as of yet unknown and requires further investigation. We note, however, that expression of GFP::LGG-1 or GFP::LGG-1(G116A) in *C. elegans* under control of the endogenous *lgg-1* promoter does not appear to cause any punctae formation in nerve-ring neurons under basal conditions[3], suggesting that LGG-1(G116A) punctae in nerve-ring neurons may represent artifacts of the overexpression conditions, rather than physiologically relevant structures.

(b) GFP::LGG-1/Atg8-positive punctae were quantified from three independent experiments in nerve-ring neurons of wild-type (WT) (N=29 animals) and *sqst-1(syb764)* animals (N=30 animals) on day 1 of adulthood expressing *rgef-1p::gfp::lgg-1.* Error bars indicate 95% CI. ****P<0.0001 by *t*-test.

(c) GFP::LGG-1/Atg8-positive punctae were quantified from three independent experiments in proximal intestinal cells of wild-type (WT) and *sqst-1(ok2892)* on day 1 of adulthood expressing *lgg-1p::gfp::lgg-1* or *lgg-1p::gfp::lgg-1(G116A)* (LGG-1(G116A). WT-LGG-1, N=82; WT-LGG-1(G116A), N=60; *sqst-1*-LGG-1, N=84, *sqst-1*-LGG-1(G116A), N=61 cells. Error bars indicate 95% CI . ns: P>0.5, ****P<0.0001, by two-way ANOVA with Tukey's multiple comparisons test. Scale bar: 10 µm.

(d) GFP::LGG-1/Atg8-positive punctae were quantified from three independent experiments in proximal intestinal cells of wild-type (WT) (N=38 cells) and *sqst-1(syb764)* animals (N=42 cells) (N=38-42, n=3) on day 1 of adulthood expressing *lgg-1p::gfp::lgg-1.* Error bars indicate 95% CI. ns: P>0.5 by *t*-test.

(e) GFP::LGG-1/Atg8-positive punctae were quantified from three independent experiments in body-wall muscle of wild-type (WT) and *sqst-1(ok2892)* on day 1 of adulthood expressing *lgg-1p::gfp::lgg-1* or *lgg-1p::gfp::lgg-1(G116A)* (LGG-1(G116A). WT-LGG-1, N=79; WT-LGG-1(G116A), N=54; *sqst-1*-LGG-1, N=74, *sqst-1*-LGG-1(G116A), N=55 animals. Error bars indicate 95% CI. ns: P>0.5, ****P<0.0001, by two-way ANOVA with Tukey's multiple comparisons test. Scale bar: 10 µm.

(f) GFP::LGG-1/Atg8-positive punctae were quantified from three independent experiments in body-wall muscle of wild-type (WT) (N=33 animals) and *sqst-1(syb764)* animals (N=55 animals) on day 1 of adulthood expressing *lgg-1p::gfp::lgg-1.* Error bars indicate 95% CI. ns: P>0.5 by *t*-test.

(g) GFP::LGG-1/Atg8-positive punctae were quantified from three independent experiments in the terminal pharyngeal bulb of wild-type (WT) and *sqst-1(ok2892)* on day 1 of adulthood expressing *lgg-1p::gfp::lgg-1* or *lgg-1p::gfp::lgg-1(G116A)* (LGG-1(G116A). WT-LGG-1, N=81; WT-LGG-1(G116A), N=69; *sqst-1*-LGG-1, N=38, *sqst-1*-LGG-1(G116A), N=73 animals. Error bars indicate 95% CI. ns: P>0.5, ****P<0.0001, by two-way ANOVA with Tukey's multiple comparisons test. Scale bar: 10 µm.

(h) GFP::LGG-1/Atg8-positive punctae were quantified from three independent experiments in the terminal pharyngeal bulb of wild-type (WT) (N=18 animals) and *sqst-1(syb764)* animals (N=22 animals) on day 1 of adulthood expressing *lgg-1p::gfp::lgg-1.* Error bars indicate 95% CI. ns: P>0.5 by *t*-test.

(i) GFP::LGG-1/Atg8-positive punctae were quantified from three independent experiments in the hypodermal seam cells of wild-type (WT) and *sqst-1(ok2892)* on day 1 of adulthood expressing *lgg-1p::gfp::lgg-1* or *lgg-1p::gfp::lgg-1(G116A)* (LGG-1(G116A). WT-LGG-1, N=135; WT-LGG-1(G116A), N=113; *sqst-1*-LGG-1, N=117, *sqst-1*-LGG-1(G116A), N=72 cells. Error bars indicate 95% CI. ns: P>0.5, **P<0.01, by two-way ANOVA with Tukey's multiple comparisons test. Scale bar: 10 µm.

(j) Autophagy flux was measured in wild-type (WT) and *sqst-1(ok2892)* animals on day 1 of adulthood expressing *lgg-1p::gfp::lgg-1*. WT and *sqst-1* animals were injected with vehicle (DMSO) or bafilomycin A1 (BafA) to block autophagy at the lysosomal acidification step. GFP::LGG-1/Atg8-positive punctae were quantified from three independent experiments in hypodermal seam cells (WT-DMSO, N=90; WT-BafA, N=69; *sqst-1*-DMSO, N=91, *sqst-1*-BafA, N=79 cells). Error bars indicate 95% CI. ns: P<0.05, **P<0.01, by two-way ANOVA with Tukey's multiple comparisons test.

As previously reported, we found that hypodermal seam cells displays a high propensity to form lipidation-independent structures[3], and that BafA injections did not increase the number of GFP::LGG-1/Atg8-positive punctae observed in *sqst-1(ok2892)* mutants. Collectively, these results are consistent with autophagy being blocked in hypodermal seam cells of *sqst-1(ok2892)* mutants, and we did not further pursue this cell type for autophagy phenotypes in this study.

Source data are provided in the Source Data file.

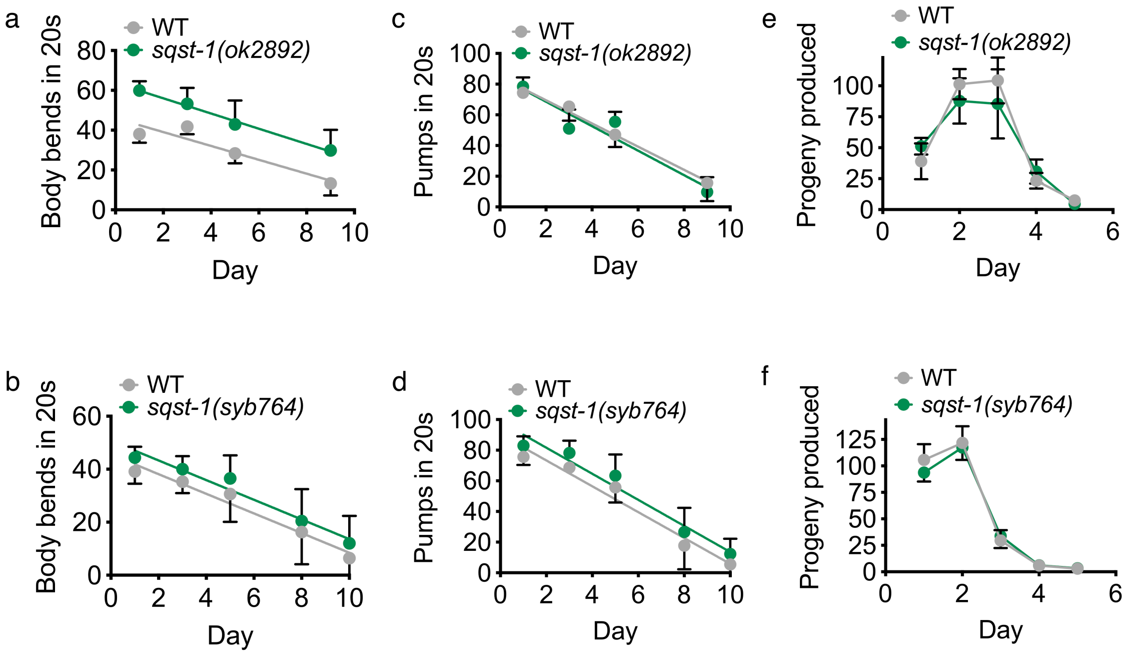

Supplementary Figure 3: Healthspan analyses of *sqst-1* mutants.

(a-b) Wild-type (WT, N2), *sqst-1(ok2892)* **(a),** and *sqst-1(syb764)* **(b)** animals were assayed from day 1 through day 10 of adulthood for thrashing ability in liquid. In such swimming assays, the number of body bends was counted for 20 s after animals were placed into a drop of M9 media. **(a)** Data are the mean ± 95% CI of N=15 animals; $P_{(m)}=0.8$; $P_{(y\text{-intercept})}<0.001$ for *sqst-1(ok2892)* by linear regression comparison. The experiment was performed at least three times with similar results. **(b)** Data are the mean ± 95% CI of N=14 animals; $P_{(m)}=0.9$; $P_{(y\text{-intercept})}<0.02$ for *sqst-1(syb764)* by linear regression comparison. The experiment was performed twice with similar results. While we currently have no explanation for this phenotype, we could speculate that the reduced number of GFP::LGG-1/Atg8-positive punctae in neurons of *sqst-1* mutants, implying reduced levels of *sqst-1*-dependent autophagy, could change the innervation or neurotransmitter release required for locomotion. More experiments are needed to address this point.

(c-d) Wild-type (WT, N2), *sqst-1(ok2892)* **(c),** and *sqst-1(syb764)* **(d)** animals were assayed from day 1 through day 10 of adulthood for pharyngeal pumping. For pharyngeal pumping assays, the number of contractions in the terminal pharyngeal bulb was counted for 20 s. **(c)** Data are the mean ± 95% CI of N=14 animals; $P_{(m)}=0.8$; $P_{(y\text{-intercept})}=0.7$ for *sqst-1(ok2892)* by linear regression comparison. This experiment was performed three times with similar results. **(d)** Data are the mean ± 95% CI of N=14 animals; $P_{(m)}=0.9$; $P_{(y\text{-intercept})}=0.09$ for *sqst-1(syb764)* by linear regression comparison. This experiment was performed twice with similar results. If no error bar is clearly visible, it was too small to be displayed.

(e-f) Wild-type (WT, N2) *sqst-1(ok2892)* **(e),** and *sqst-1(syb764)* **(f)** were assayed for progeny production. For reproductive span, the number of eggs and larvae produced per day per animal were counted. **(e)** Data are the mean ± 95% CI of N=10 animals. P>0.05 for all time points by two-way ANOVA with Tukey's multiple comparisons. This experiment was repeated twice with similar results. Our results are in contrast to a previous report listing a reduced brood size in *sqst-1(ok2892)* mutants[4]. This discrepancy could be either due to different laboratory conditions or the elimination of this phenotype after multiple backcrossing to wild-type animals. **(f)** Data are the mean ± 95% CI of N=24 animals. P>0.05 for all time points by two-way ANOVA with Tukey's multiple comparisons. This experiment was repeated twice with similar results.

Source data are provided in the Source Data file.

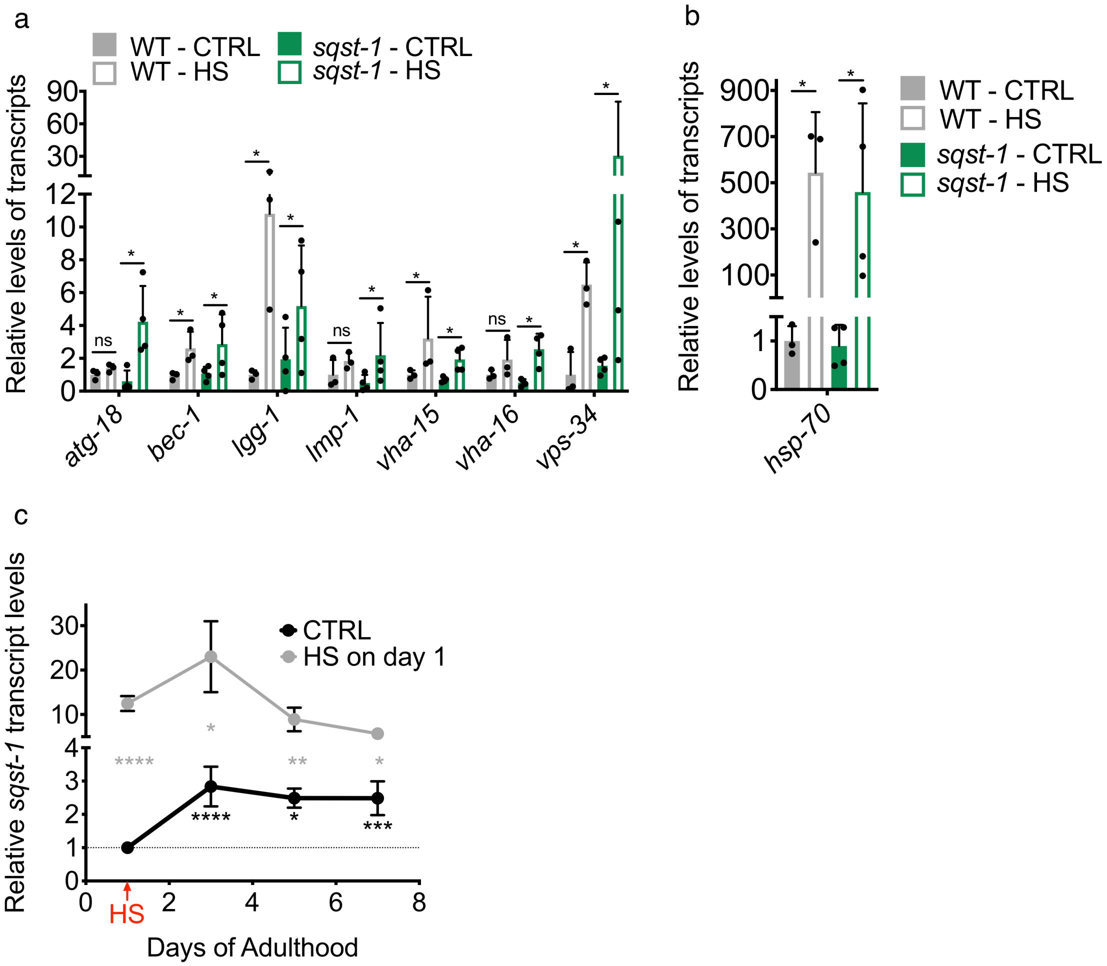

Supplementary Figure 4: Gene-expression analysis upon heat shock.

(a-b) Transcript levels of genes involved in **(a)** various steps of the autophagy process, and **(b)** of heat shock protein *hsp-70* in wild-type (WT) and *sqst-1(ok2892)* (*sqst-1*) animals on day 1 of adulthood maintained under control conditions (CTRL) or subjected to heat shock for 1 h at 36 °C (HS). Data are the mean ± SD of three biological replicates, each with three technical replicates, and were normalized to the mean expression levels of levels of housekeeping genes *nhr-23*, *ama-1*, and *cdc-42*. ns: P>0.05, *P<0.05 by multiple *t*-tests.

(c) Transcript levels of *sqst-1* in wild-type (N2) animals maintained under control conditions (CTRL) or subjected to heat shock (HS) for 1 h at 36 °C on day 1 of adulthood (HS on day 1) on day 1, 3, 5 and 7 of adulthood. Data are the mean ± SEM of three biological replicates, each with three technical replicates, and are normalized to the mean expression levels of three housekeeping genes and relative to samples of CTRL animals on day 1 of adulthood. *P<0.05, **P<0.01, ***P<0.001, ****P<0.0001 by one-way ANOVA with Dunnett's multiple comparisons test for comparison of age-related increase of *sqst-1* mRNA levels (black asterisks), and by two-way ANOVA with Tukey's multiple comparisons test for comparison of heat shock-dependent increase of *sqst-1* mRNA levels (grey asterisks).

Source data are provided in the Source Data file.

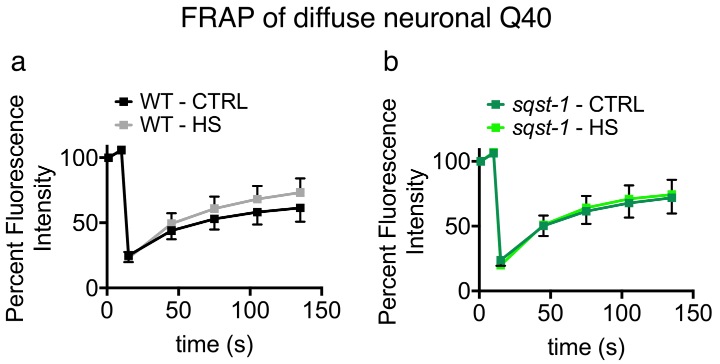

Supplementary Figure 5: Analysis of diffuse portion of neuronal PolyQ aggregation in *sqst-1(ok2892)* mutants.

FRAP measurements of the diffuse portion of neuronal PolyQ *(rgef-1p::Q40::yfp)* on day 8 of adulthood in wild-type (WT) **(a)** and *sqst-1(ok2892)* (*sqst-1*) **(b)** animals maintained under control conditions (CTRL) or subjected to HS (1 h at 36 °C) on day 1 of adulthood (HS). Fluorescence signal bleached after 10 and fluorescence recovery was measured for 2 min. The average % of fluorescence intensity was quantified from three independent experiments in WT-CTRL, N=32; WT-HS, N=30; *sqst-1*-CTRL, N=32, *sqst-1*-HS, N=31 animals. Error bars indicate 95% CI. All time points are P>0.05 by two-way ANOVA with Sidak's multiple comparisons test. P>0.05 for all timepoints when comparing WT-CTRL and *sqst-1*-CTRL by two-way ANOVA with Sidak's multiple comparisons test. Source data are provided in the Source Data file.

Supplementary Figure 6

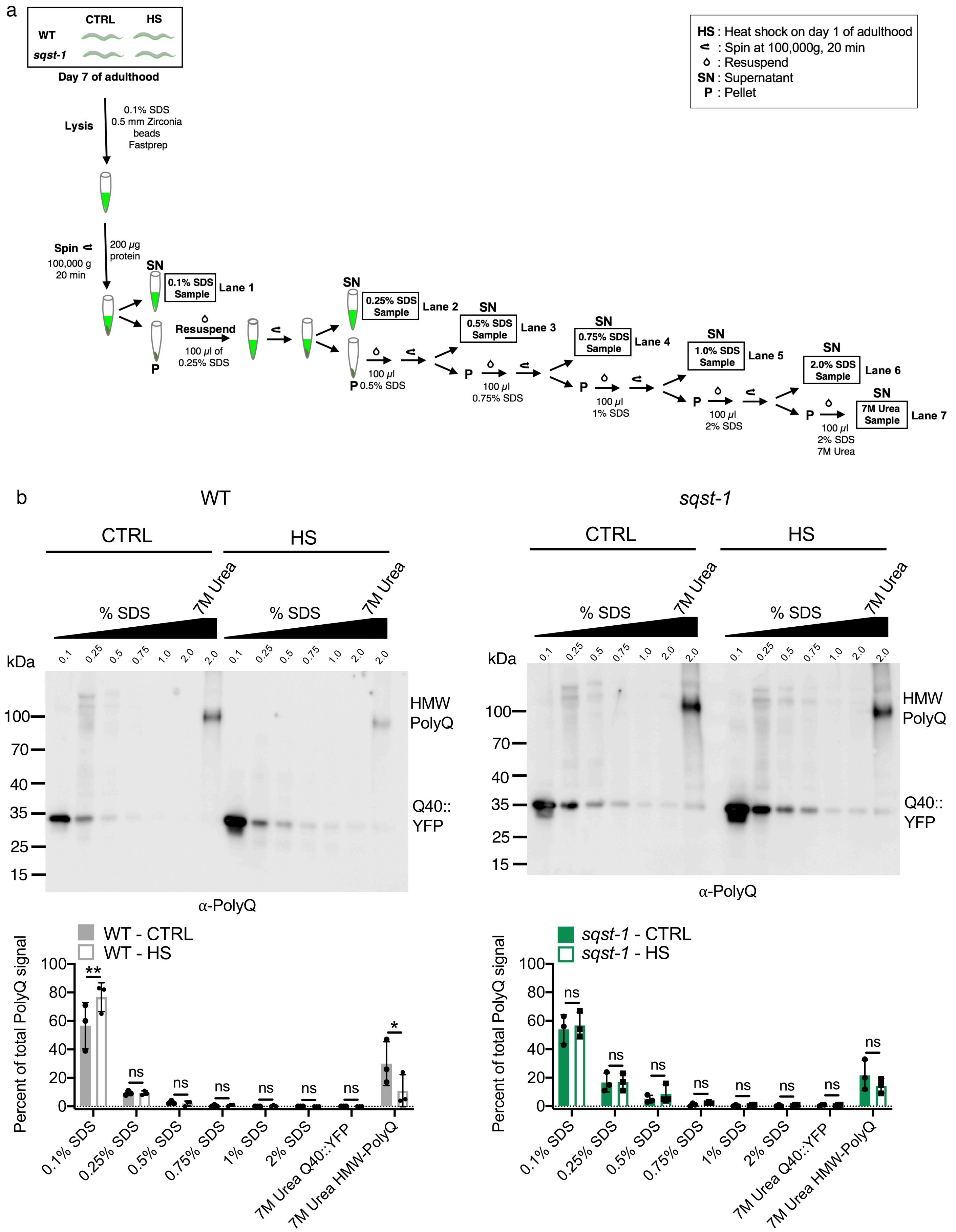

Supplementary Figure 6: Analysis of neuronal PolyQ aggregation in *sqst-1(ok2892)* mutants.

(a) Schematic of differential detergent extraction experiment. 1-day old WT and *sqst-1(ok2892)* animals expressing neuronal Q40 proteins *(rgef-1p::Q40::yfp)* were either subjected to a 1 h heat shock at 36°C, or kept at control conditions. After the heat shock, animals were placed on media plates containing FuDR to prevent progeny production. Animals were aged until day 7 of adulthood and effects of heat shock on aggregate formation was confirmed microscopically. 100 μl of worm pellet (~30,000 animals) were lysed by homogenization using a Fastprep homogenizer (3 x 6000 rpm, 45 s, with 30 s pause). 200 μg of protein lysate was subjected to ultracentrifugation (100,000 g, 20 min, 4°C). The supernatant was transferred to a fresh tube and kept for Western blot analysis. The pellet was resuspended in 100 μl lysis buffer with increasing SDS concentration (0.25%, 0.5%, 0.75%, 1.0%, or 2% SDS), followed by another centrifugation step (100,000 g, 20 min, 4°C). After the last centrifugation step, the pellet was dissolved in 100 μl urea buffer. The samples were loaded in corresponding order on an SDS-gel (Lane 1-7, as indicated in the figure).

(b) Representative western blot of samples collected in differential detergent extraction. Quantification of the relative fraction of protein extracted with the corresponding concentration of SDS and urea buffer, expressed as a percentage of the total detected PolyQ signal. Error bars indicate SD from three independent experiments. ns: $P>0.05$, *$P<0.05$, **$P<0.01$, two-way ANOVA with Sidak's multiple comparisons test. $P>0.05$ for all timepoints when comparing WT-CTRL and *sqst-1*-CTRL by two-way ANOVA with Sidak's multiple comparisons test. Source data are provided in the Source Data file.

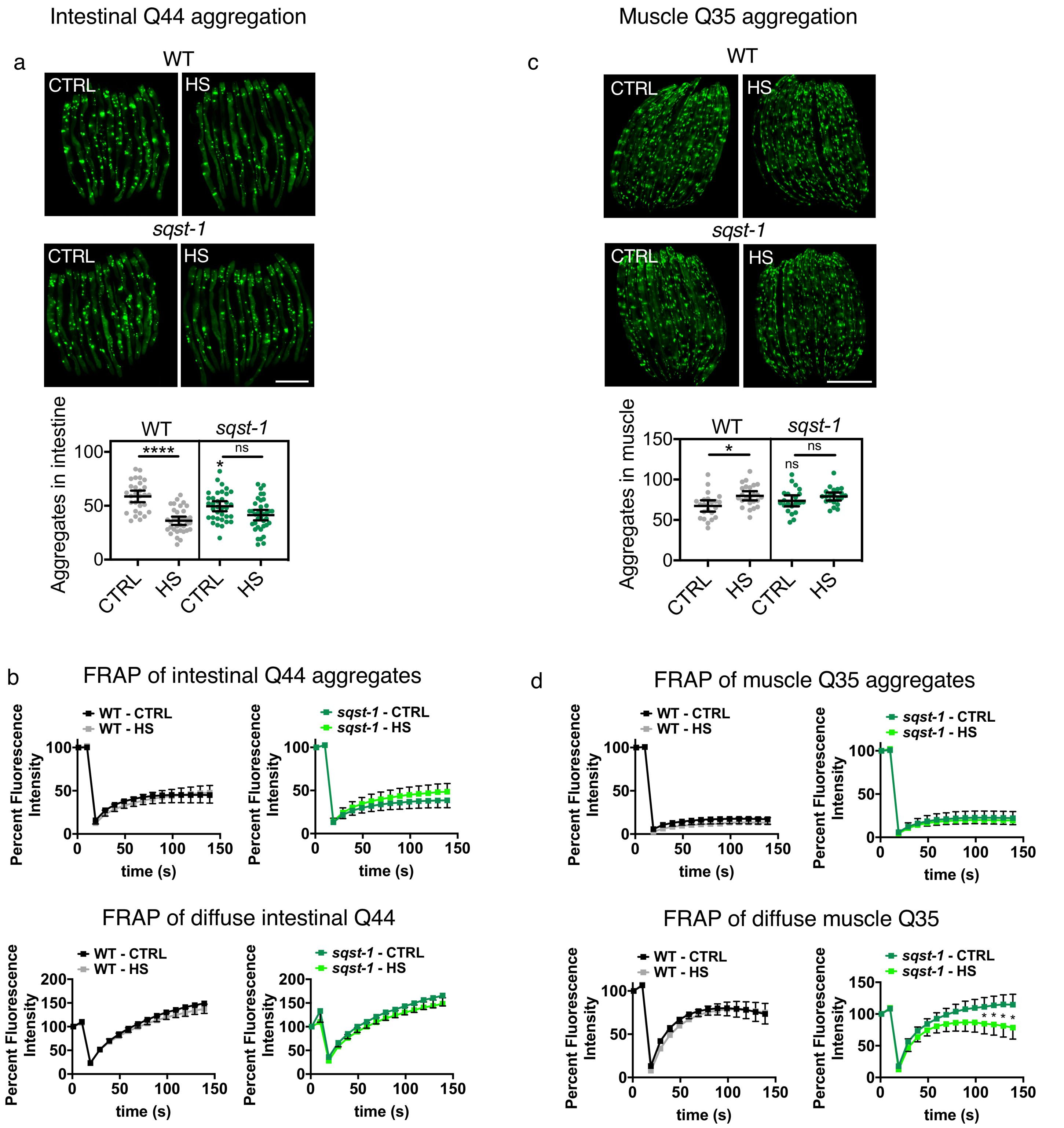

Supplementary Figure 7: Analysis of tissue-specific PolyQ aggregation in *sqst-1(ok2892)* mutants.

(a) Intestinal PolyQ *(vha-6p::Q44::yfp)* aggregates detected on day 5 of adulthood in wild-type (WT) and *sqst-1(ok2892)* (*sqst-1*) animals maintained under control conditions (CTRL) or subjected to HS (1 h at 36 °C) on day 1 of adulthood (HS). Scale bar 200 μm. The number of neuronal PolyQ aggregates were quantified from three independent experiments in WT-CTRL, N=29; WT-HS, N=35; *sqst-1*-CTRL, N=35, *sqst-1*-HS, N=37 animals.

Error bars indicate 95% CI. ns: P>0.05, *P<0.05, ****P<0.0001, by two-way ANOVA with Tukey's multiple comparisons test.

(b) FRAP measurements of the aggregated and diffuse portion of intestinal PolyQ *(vha-6p::Q44::yfp)* on day 3 of adulthood in wild-type (WT) and *sqst-1(ok2892)* (*sqst-1*) animals maintained under control conditions (CTRL) or subjected to HS (1 h at 36 °C) on day 1 of adulthood (HS). Fluorescence signal was bleached after 20 s and fluorescence recovery was measured for 2 min. The average % of fluorescence intensity was quantified from three independent experiments in WT-CTRL, N=32; WT-HS, N=28; *sqst-1*-CTRL, N=32, *sqst-1*-HS, N=28 animals. Error bars indicate 95% CI. All measurements are P>0.05 by two-way ANOVA with Sidak's multiple comparisons test.

(c) Muscle PolyQ *(unc-54p::Q35::yfp)* aggregates were detected and quantified on day 3 of adulthood in wild-type (WT) and *sqst-1(ok2892)* (*sqst-1*) animals maintained under control conditions (CTRL) or subjected to hormetic heat shock (1 h at 36 °C) on day 1 of adulthood (HS). Scale bar 200 μm. The number of neuronal PolyQ aggregates were quantified from two independent experiments in WT-CTRL, N=22; WT-HS, N=24; *sqst-1*-CTRL, N=21, *sqst-1*-HS, N=21 animals. Error bars indicate 95% CI. ns: P>0.4, *P<0.02, by two-way ANOVA with Tukey's multiple comparisons test.

(d) FRAP measurements of the aggregated and diffuse portion of muscle PolyQ *(unc-54p::Q35::yfp)* on day 3 of adulthood in wild-type (WT) and *sqst-1(ok2892)* (*sqst-1*) animals maintained under control conditions (CTRL) or subjected to HS (1 h at 36 °C) on day 1 of adulthood (HS). Fluorescence signal bleached after 10 s and fluorescence recovery was measured for 2 min. The average % of fluorescence intensity was quantified from three independent experiments in WT-CTRL, N=27; WT-HS, N=26; *sqst-1*-CTRL, N=32, *sqst-1*-HS, N=26 animals. Error bars indicate 95% CI. All measurements are P>0.05 by two-way ANOVA with Sidak's multiple comparisons test unless otherwise indicated (*P<0.05).

Source data are provided in the Source Data file.

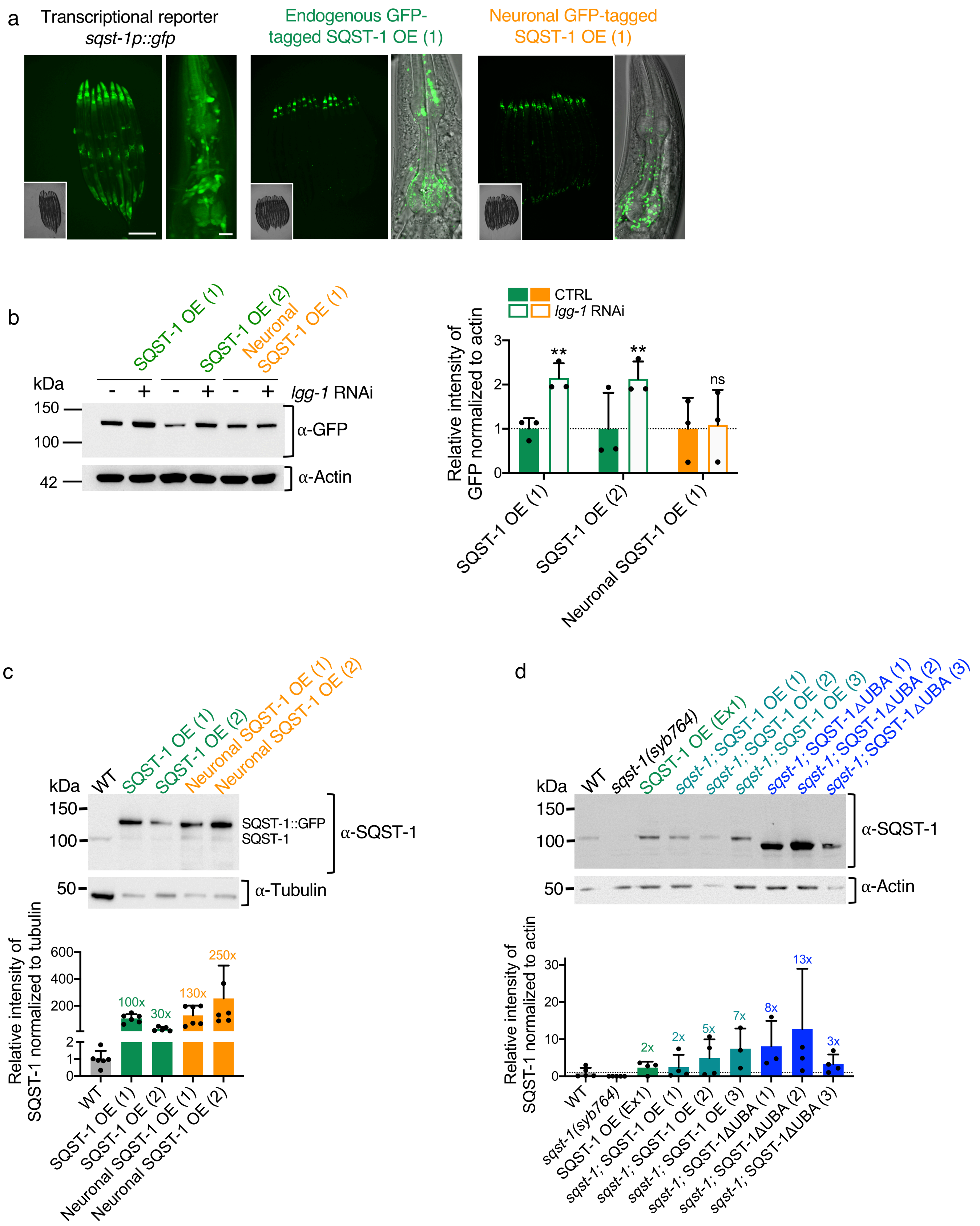

Supplementary Figure 8: Quantification of SQST-1 overexpression in transgenic strains.

(a) GFP expression in animals expressing the transcriptional reporter *sqst-1p::gfp*, genomically encoded GFP-tagged SQST-1 (*sqst-1(syb940[sqst-1::gfp]), sqst-1p::sqst-1::gfp* (one of two independently integrated lines

denoted as SQST-1 OE (1) and (2) throughout manuscript, see **Supplemental Data 4** for details), or *rgef-1p::sqst-1p::gfp* (one of two independently integrated lines denoted as neuronal SQST-1 OE (1) and (2) throughout the manuscript) on day 1 of adulthood. Exposure time, 500 ms. Scale bar, 200 μm and 10 μm for close-up of head region. Inserts show DIC picture of the aligned animals.

(b) Representative Western blot of SQST-1-overexpressing animals (*sqst-1p::sqst-1::gfp*, SQST-1 OE (1) and SQST-1 OE (2)), and neuronal SQST-1 overexpressing animals (*rgef-1p::sqst-1::gfp*, Neuronal SQST-1 OE (1)) fed bacteria expressing either empty vector (CTRL) or dsRNA targeting autophagy-related gene *lgg-1/ATG8* (*lgg-1* RNAi) for four generations to block autophagy. 50 animals were handpicked for each strain. Antibodies against GFP (α-GFP) and actin (α-Actin) were used. Quantification of the abundance of SQST-1::GFP upon reduction of *lgg-1/ATG8* was normalized to actin and is depicted as relative to control conditions from three independent experiments. Error bars indicate SD. We note that SQST-1 abundance significantly increased upon reducing the levels of *lgg-1/ATG8* using RNAi in animals overexpressing GFP-tagged::SQST-1 from integrated loci. This indicates that the tagged SQST-1 protein is functional in that it can serve as a substrate for autophagy[3]. In contrast, there was no increase in the abundance of SQST-1 after subjecting animals neuronally overexpressing GFP-tagged::SQST-1 to *lgg-1/ATG8* using RNAi, presumably because neurons are largely refractory to RNAi[5].

(c) Representative Western blot of wild-type (WT, N2) animals, SQST-1-overexpressing animals (*sqst-1p::sqst-1::gfp*, SQST-1 OE (1) and SQST-1 OE (2)), and neuronal SQST-1 overexpressing animals (*rgef-1p::sqst-1::gfp*, Neuronal SQST-1 OE (1)). 10 animals were handpicked for each strain, except for WT for which ~500 animals were washed of multiple media plates. Antibodies against *C. elegans* SQST-1 (α-SQST-1) and tubulin (α-Tubulin) were used. Quantification of the abundance of SQST-1::GFP normalized to tubulin and relative to WT was from six independent experiments. Error bars indicate SD. We note that the SQST-1 expression levels in wild-type (N2) animals as well as in SQST-1-overexpressing animals are prone to fluctuation, presumably because of the constant degradation of SQST-1 by autophagy.

(d) Representative Western blot of wild-type animals (WT, N2, N~200 animals), *sqst-1(syb764)* mutants (N~200 animals), transgenic animals carrying extrachromosomal arrays expressing untagged SQST-1 in wild-type (SQST-1 OE (Ex1) or *sqst-1(syb764)* backgrounds (*sqst-1;* SQST-1 OE (1-3)) or untagged SQST-1ΔUBA (*sqst-1;* SQST-1ΔUBA (1-3)) (N=50 animals per sample), using antibodies against *C. elegans* SQST-1 (α-SQST-1) and actin (α-Actin). Quantification of the abundance of SQST-1 was normalized to actin and relative to WT was from three independent experiments. Error bars indicate SD.

Source data for b-d are provided in the Source Data file.

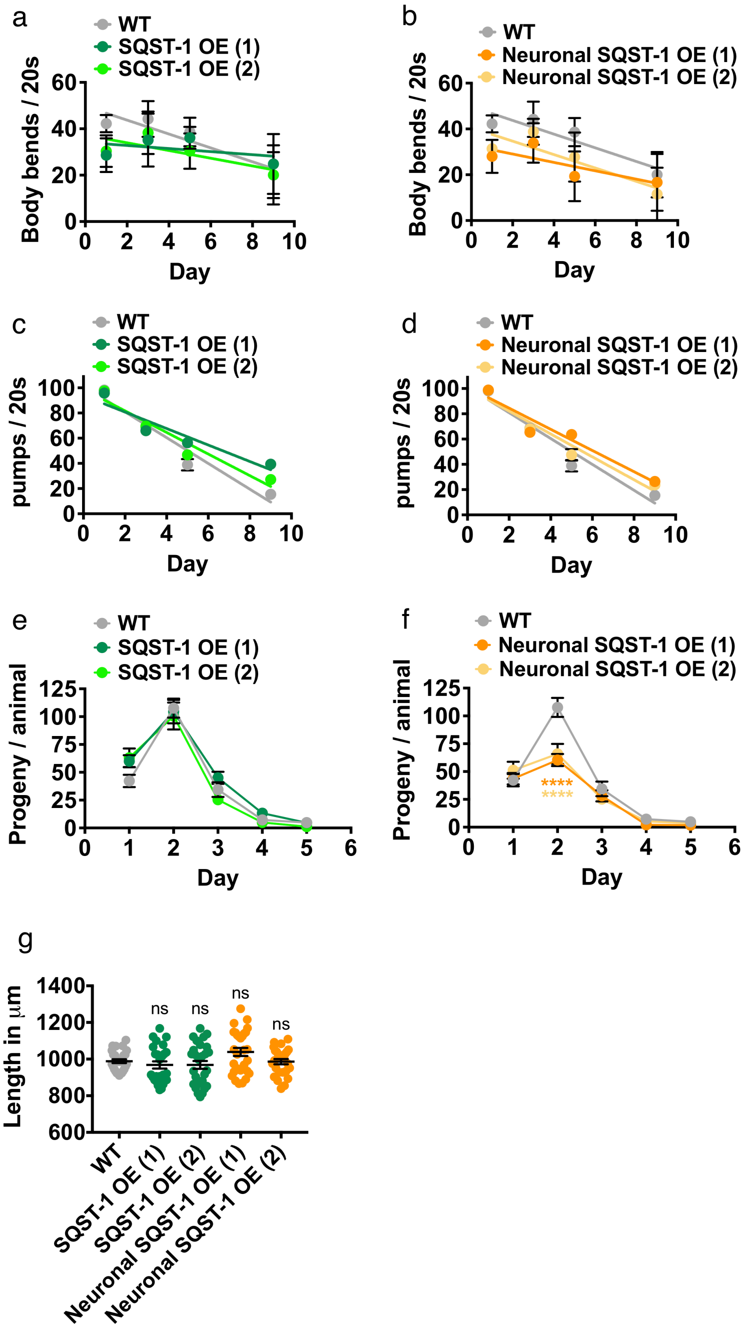

Supplementary Figure 9: Healthspan analyses of *C. elegans* strains overexpressing SQST-1.

Wild-type animals (WT), transgenic animals stably overexpressing either *sqst-1p::sqst-1::gfp* (SQST-1 OE (1) and (2)) or *rgef-1p::sqst-1::gfp* (Neuronal SQST-1 OE (1) and (2)) were assayed from day 1 through day 9 of adulthood for **(a-b)** thrashing ability in liquid, **(c-d)** pharyngeal pumping, **(e-f)** reproductive span, and **(g)** body length.

(a-b) For swimming assays, the number of body bends was counted for 20 s after animals were placed into a drop of M9 media. Data are the mean ± SEM of N=10 animals per strain; **(a)** $P_{(m)}<0.0001$, **(b)** $P_{(m)}=0.7$, $P_{(y\text{-intercept})}=0.04$ by linear regression comparison. This experiment was repeated at least three times with similar results.

(c-d) For pharyngeal pumping assays, the number of contractions in the terminal pharyngeal bulb was counted for 20 s. Data are the mean ± SEM of N=20 animals per strain; **(c)** $P_{(m)}=0.4$; $P_{(y\text{-intercept})}=0.5$, **(d)** $P_{(m)}=0.7$; $P_{(y\text{-}}$

$_{intercept)}$=0.5 by linear regression comparison. This experiment was repeated three times with similar results. If no error bar is visible, it was too small to be displayed.

(e-f) For reproductive span, the number of eggs and larvae produced per day per animal were counted. Data are the mean ± SEM of N=10 animals per strain. ****P<0.0001 and P>0.05 for all other time points by two-way ANOVA with Tukey's multiple comparisons. We note that SQST-1 overexpression under the control of the pan-neuronal promoter, but not the endogenous promoter, lead to a decrease in the number of progeny production, indicating a possible trade-off between longevity and progeny production in animals overexpressing SQST-1 in neurons. This experiment was repeated twice with similar results. If no error bar is visible, it was too small to be displayed.

(g) Body length was measured of animals (N=26-29, n=2) that were anesthetized with NaN_3 and aligned under a stereoscope. WT, N=28; SQST-1 OE (1), N=26; SQST-1 OE (2), N=28; Neuronal SQST-1 OE (1), N=29; Neuronal SQST-1 OE (2), N=27 animals. Error bars indicate SEM. ns: P>0.05, by one-way ANOVA with Dunnett's multiple comparisons.

Source data are provided in the Source Data file.

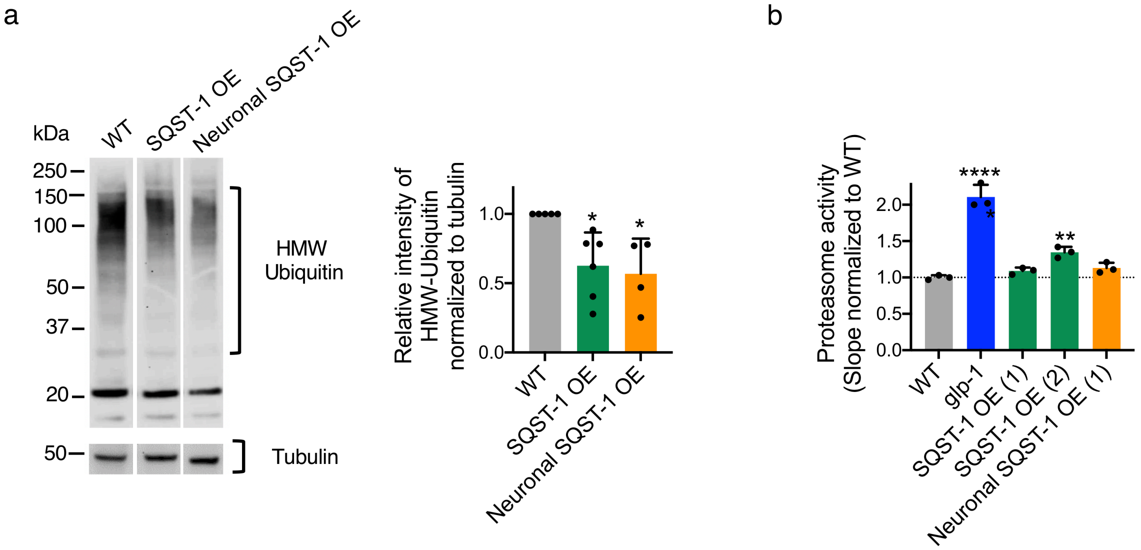

Supplementary Figure 10: SQST-1-overexpressing animals have reduced levels of ubiquitinated proteins, but only modestly increased proteasome activity.

(a) Representative Western blot of wild-type (WT), SQST-1-overexpressing (*sqst-1p::sqst-1::gfp)* (SQST-1 OE) and neuronal SQST-1-overexpressing (*rgef-1p::sqst-1::gfp)* (Neuronal SQST-1 OE) animals on day 1 of adulthood using antibodies against ubiquitin, indicating high molecular weight (HMW) ubiquitin, free ubiquitin and tubulin. 50 animals were handpicked for each strain. Quantification of the relative abundance of HMW-ubiquitin normalized to tubulin of three independent experiments. Error bars indicate SD. *P<0.05, by one-way ANOVA with Dunnett's multiple comparisons test.

(b) Chymotrypsin-like proteasome activity was measured in wild-type (WT, N2) animals, *glp-1(e2144)* mutants as positive control for elevated proteasome activity[6], SQST-1-overexpressing (*sqst-1p::sqst-1::gfp)* (SQST-1 OE (1) and SQST-1 OE (2)), and neuronal SQST-1-overexpressing (*rgef-1p::sqst-1::gfp)* (Neuronal SQST-1 OE(1)) animals on day 3 of adulthood after animals were placed on media plates containing FuDR to prevent progeny production from day 0 of adulthood on (see Methods). Slopes of the fluorescence intensity caused by cleavage of an in-vitro substrate is calculated and displayed relative to WT. Error bars indicate SD. of three biological samples. **P<0.01, ****P<0.0001 and P>0.05 (no symbol) by one-way ANOVA with Dunnett's multiple comparisons test. This experiment was repeated four times with similar results.

Source data are provided in the Source Data file.

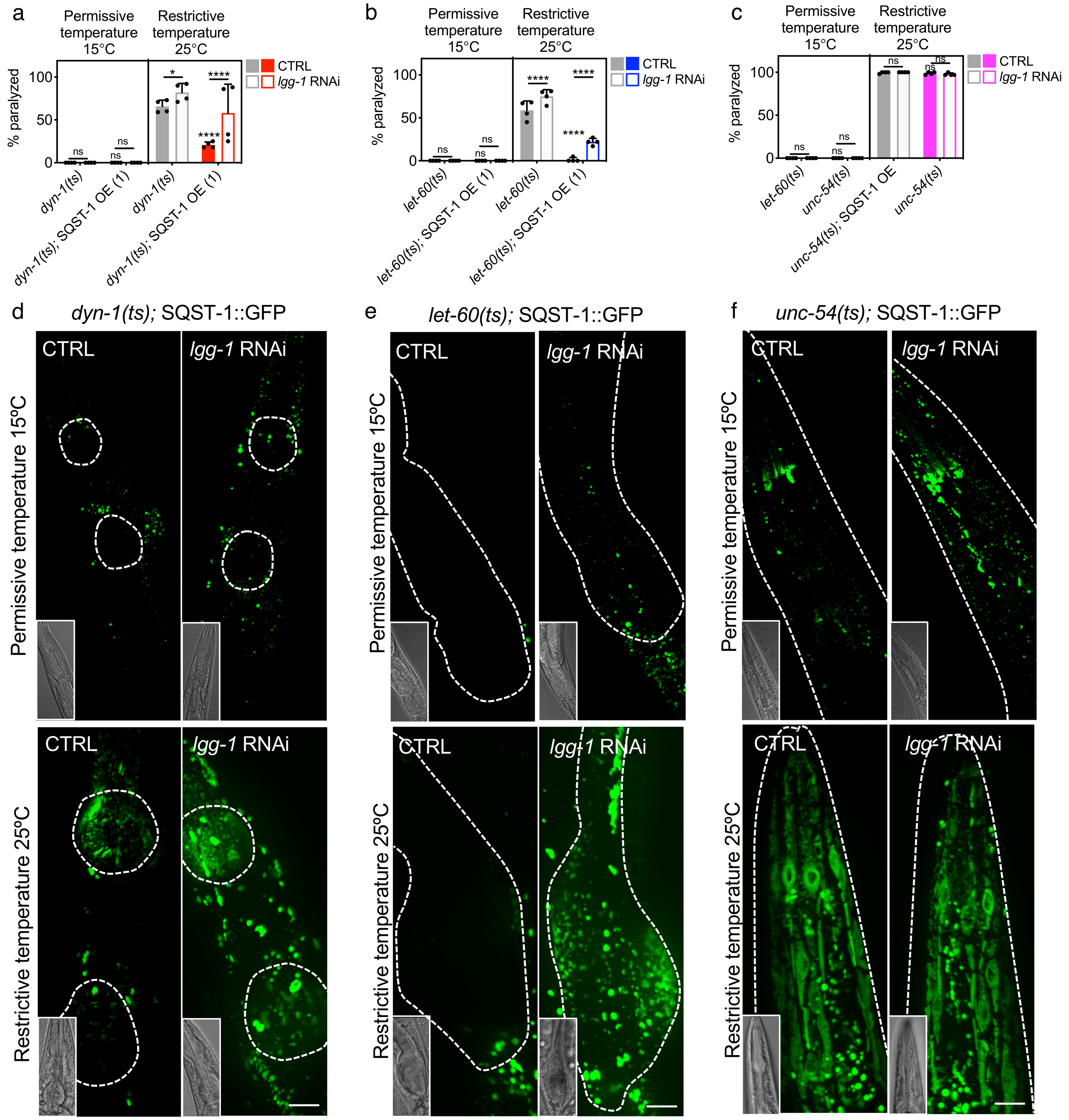

Supplementary Figure 11: SQST-1/p62 overexpression improves proteostasis of temperature-sensitive protein-folding mutants.

(a-c) Paralysis was assessed in animals carrying temperature-sensitive (ts) missense mutations and overexpressing *sqst-1p::sqst-1::gfp* (SQST-1 OE). Animals were fed bacteria expressing empty vector (CTRL) or dsRNA targeting autophagy gene *lgg-1/Atg8* from hatching on, and were either raised and kept at the permissive temperature of 15°C or raised at 20°C until day 0 of adulthood and then transferred to the restrictive temperature of 25°C. On day 3 of adulthood, paralysis was scored. **(a)** Neuronal dynamin GTPase *dyn-1(ts)* (n=4 plates); **(b)** Intestinal *let-60(ts)* (Ras) (n=4 plates); **(c)** body-wall muscle *unc-54(ts)* (myosin) (n=4 plates). Error bars indicate SD. Where error bars are missing, 0% or 100% paralysis was observed on all plates. ns

P>0.05, ****P<0.0001 by two-way ANOVA with Tukey's multiple comparisons. This experiment was performed at least four times with similar results. Source data for a-c are provided in the Source Data file.

(d-f) Tissue-specific GFP expression in animals carrying temperature-sensitive (ts) missense mutations and expressing *sqst-1p::sqst-1::gfp.* Animals were fed bacteria expressing empty vector (CTRL) or dsRNA targeting autophagy gene *lgg-1/Atg8* from day 0 of adulthood on, were either raised and kept at the permissive temperature of 15°C, or raised at 20°C until day 0 of adulthood and then transferred to the restrictive temperature of 25°C. Animals were imaged on day 2 of adulthood. Scale bar, 10 µm. **(d)** Image of head region of *dyn-1(ts);* SQST-1::GFP animals with outline of pharyngeal bulbs. **(e)** Image of outlined distal intestines of *let-60(ts);* SQST-1::GFP animals. (**f**) Image of body-wall muscles in outlined head region of *unc-54(ts);* SQST-1::GFP animals. Inserts show DIC picture of the animal.

Supplementary Figure 12

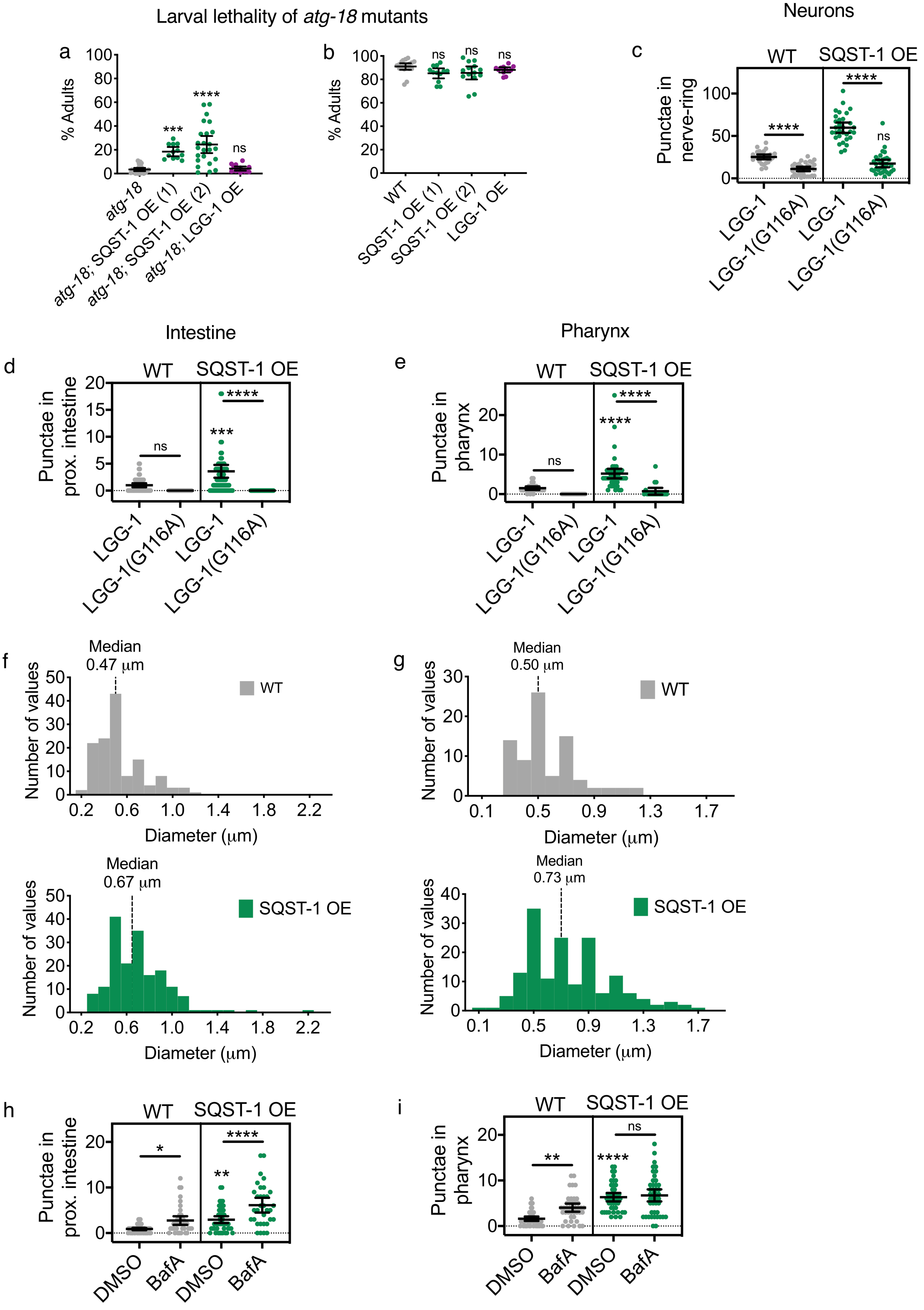

Supplementary Figure 12: Additional characterization of autophagy in SQST-1-overexpressing animals.

(a-b) Percent of animals that developed into adulthood in **(a)** *atg-18(gk378)* (*atg-18*) mutants, or **(b)** wild-type (WT) animals expressing integrated *sqst-1p::sqst-1::gfp* (SQST-1 OE (1) and (2)) or *lgg-1p::gfp::lgg-1* (LGG-1 OE). A defined number of eggs were transferred onto media plates with OP50 bacteria as a food source and the number of adults present on each NGM was counted after 4 days. (**a**) *atg-18,* N=24; *atg-18,* SQST-1 OE (2), N=24; *atg-18,* SQST-1 OE (1), N=12; *atg-18,* LGG-1 OE, N=14 plates. (**b**) WT, N=19; SQST-1 OE (2), N=14; SQST-1 OE (1), N=12; LGG-1 OE, N=12 plates. Error bars indicate 95% CI. ns: P<0.05, ****P<0.0001, by one-way ANOVA with Tukey's multiple comparisons test.

(c) GFP::LGG-1/Atg8-positive punctae were quantified from three independent experiments in nerve-ring neurons of wild-type (WT) and SQST-1 OE animals expressing *rgef-1p::gfp::lgg-1* (LGG-1) or *rgef-1p::gfp::lgg-1((*LGG-1(*G116A)).* WT-LGG-1, N=27; WT-LGG-1(G116A), N=31; SQST-1 OE-LGG-1, N=31, SQST-1 OE-LGG-1(G116A), N=32 animals. ****P<0.0001, ns: P<0.05 by two-way ANOVA with Tukey's multiple comparisons test. We note that LGG-1(G116A) expressed specifically in neurons formed some lipidation-independent structures in neurons (see figure legend of **Supplementary Fig. 2** for further discussion), but this fraction of lipidation-independent GFP::LGG-1/Atg8-positive structures was not affected by SQST-1 overexpression.

(d-e) GFP::LGG-1/Atg8-positive punctae were quantified from three independent experiments in wild-type (WT) and SQST-1-overexpressing (SQST-1 OE) (*sqst-1p::sqst-1+rol-6;* extrachromosomal array) animals on day 1 of adulthood expressing *lgg-1p::gfp::lgg-1* (LGG-1) or *lgg-1p::gfp::lgg-1(G116A)* (LGG-1(G116A)) in in **(c)** proximal intestinal cells (WT-LGG-1, N=56; WT-LGG-1(G116A), N=35; SQST-1 OE-LGG-1, N=88, SQST-1 OE-LGG-1(G116A), N=46 cells) or **(d)** terminal pharyngeal bulb (WT-LGG-1, N=26; WT-LGG-1(G116A), N=20; SQST-1 OE-LGG-1, N=46, SQST-1 OE-LGG-1(G116A), N=25 animals). Error bars indicate 95% CI. ns: P<0.05, ***P<0.001, ****P<0.0001, by two-way ANOVA with Tukey's multiple comparisons test. The body-wall muscle was difficult to detect in these strains and could not be analyzed.

(f-g) Histograms of GFP::LGG-1/Atg8-positive punctae of wild-type and SQST-1-overexpressing (SQST-1 OE) animals with indicated median diameter binned by their diameter in **(e)** proximal intestinal cells (WT, N=133; SQST-1 OE, N=174 GFP::LGG-1/Atg8 structures), or **(f)** terminal pharyngeal bulb (WT, N=81; SQST-1 OE, N=161 GFP::LGG-1/Atg8 structures). See **Supplementary Table 5** for statistical comparison.

(h-i) Autophagy flux was measured in WT and SQST-1 OE (*sqst-1p::sqst-1+rol-6;* extrachromosomal array) animals on day 1 of adulthood expressing *lgg-1p::gfp::lgg-1*. WT and SQST-1-overexpressing animals were injected with vehicle (DMSO) or bafilomycin A1 (BafA) to block autophagy at the lysosomal acidification step. GFP::LGG-1/Atg8-positive punctae were quantified in **(g)** proximal intestinal cells (WT-DMSO, N=52; WT-BafA, N=40; SQST-1 OE-DMSO, N=50, SQST-1 OE-BafA, N=33 cells) and **(h)** terminal pharyngeal bulb (WT-DMSO, N=46; WT-BafA, N=41; SQST-1 OE-DMSO, N=45, SQST-1 OE-BafA, N=44 animals). Error bars indicate 95% CI. ns: P<0.05, *P<0.05, **P<0.01, ****P<0.0001, by two-way ANOVA with Tukey's multiple comparisons test. The body-wall muscle was difficult to see in these strains and could not be analyzed.

Source data are provided in the Source Data file.

2. Supplementary Tables

Supplementary Table 1: Thermorecovery analyses of *sqst-1(ok2892)* animals after hormetic heat shock.

Exp	Strain	Heat shock conditions	% alive ± SEM	N	*P*-value
1	WT	-	11±4	106	
	sqst-1(ok2892)	-	12±5	101	ns
2	WT	-	4±0	100	
	sqst-1(ok2892)	-	9±4	101	ns
3	WT	-	48±4	86	
	sqst-1(ok2892)	-	43±2	68	ns
4	WT	-	18±2	104	
	sqst-1(ok2892)	-	18±3	91	ns
5	WT	-	43±3	101	
	WT	HS: 1 h, 36°C, d1; TR: d4, 7 h	61±5	90	ns
	sqst-1(ok2892)	-	30±10	100	ns
	sqst-1(ok2892)	HS: 1 h, 36°C, d1; TR: d4, 7 h	14±4	86	ns (vs *sqst-1* CTRL)
6[1]	WT	-	11±3	91	
	WT	HS: 1 h, 36°C, d1; TR: d3, 7 h	54±6	95	***
	sqst-1(ok2892)		16±5	79	ns
	sqst-1(ok2892)	HS: 1 h, 36°C, d1; TR: d3, 7 h	20±4	86	ns (vs *sqst-1* CTRL)
7	WT	-	31±8	97	
	WT	HS: 1 h, 36°C, d1; TR: d3, 7 h	67±3	101	**
	sqst-1(ok2892)	-	65±8	92	**
	sqst-1(ok2892)	HS: 1 h, 36°C, d1; TR: d3, 7 h	73±4	88	ns (vs *sqst-1* CTRL)

Supplementary Table 1: Survival analysis of wild-type (WT, N2) and *sqst-1(ok2892)* animals maintained under control conditions or subjected to 1 h of hormetic heat shock (HS: 36°C) on the day 1 of adulthood (d1). Thermorecovery (TR) was tested on the indicated days of adulthood by incubating animals at 36°C for 7 h followed by ~20 h of recovery. Exp: experiment number (in chronological order); SEM: standard error of the mean; N: number of animals analyzed (see Methods). The mean ± SEM percentage survival of experimental and control groups was analyzed using Student's *t*-test, or two-way ANOVA. ns: $P > 0.05$, *$P < 0.05$, ** $P < 0.01$, *** $P < 0.001$. [1]: data depicted in **Fig. 2a** (experiment picked randomly for display).

Supplementary Table 2: Lifespan analyses of *sqst-1(ok2892)* and PolyQ-expressing animals after hormetic heat shock.

Exp	Strain	Heat shock	MLS (days)	N	% MLS	*P* value
***sqst-1(ok2892)* mutants**						
1[1]	WT		14.5	126/132		
	WT	1 h, 36°C, d1	19.2	111/133	+32% (vs WT- CTRL)	<0.0001
	sqst-1(ok2892)		14.2	116/124	-2% (vs WT-CTRL)	0.33
	sqst-1(ok2892)	1 h, 36°C, d1	12.6	106/116	-12% (vs *sqst-1*-CTRL)	0.004
2	WT		14.9	112/129		
	WT	1 h, 36°C, d1	17.9	99/128	+20% (vs WT-CTRL)	<0.0001
	sqst-1(ok2892)		15.0	101/128	0% (vs WT-CTRL)	0.54
	sqst-1(ok2892)	1 h, 36°C, d1	15.1	84/125	+1% (vs *sqst-1*-CTRL)	0.53
3	WT		15.4	117/121		
	WT	1 h, 36°C, d1	18.7	101/120	+21%(vs WT-CTRL)	<0.0001
	sqst-1(ok2892)		15.2	96/124	-1% (vs WT-CTRL)	0.46
	sqst-1(ok2892)	1 h, 36°C, d1	14.1	109/132	-7% (vs *sqst-1*-CTRL)	0.56
**Neuronal PolyQ-expressing animals**						
1	*rgef-1p::Q40::yfp*		8.0	110/125		
	rgef-1p::Q40::yfp	1 h, 36°C, d1	13.1	109/121	+65% (vs CTRL)	<0.0001
	sqst-1(ok2892); rgef-1p::Q40::yfp		9.1	118/121	+15% (vs CTRL)	0.01
	sqst-1(ok2892); rgef-1p::Q40::yfp	1 h, 36°C, d1	10.3	111/122	+13% (vs *sqst-1*- CTRL)	0.02
2	*rgef-1p::Q40::yfp*		10.3	107/121		
	rgef-1p::Q40::yfp	1 h, 36°C, d1	13.4	102/120	+31% (vs CTRL)	<0.0001
	sqst-1(ok2892); rgef-1p::Q40::yfp		13.1	122/126	+28% (vs CTRL)	0.01
	sqst-1(ok2892); rgef-1p::Q40::yfp	1 h, 36°C, d1	14.1	110/116	+8 % (vs *sqst-1*- CTRL)	0.02
**Intestinal PolyQ-expressing animals**						
1	*vha-6p::Q44::yfp*		11.7	121/126		
	vha-6p::Q44::yfp	1 h, 36°C, d1	17.0	107/116	+46% (vs CTRL)	<0.0001
	sqst-1(ok2892); vha-6p::Q44::yfp		12.6	125/128	+8% (vs CTRL)	0.26
	sqst-1(ok2892); vha-6p::Q44::yfp	1 h, 36°C, d1	14.9	118/122	+18% (vs *sqst-1*-CTRL)	<0.0001
2	*vha-6p::Q40::yfp*		13.3	131/132		
	vha-6p::Q40::yfp	1 h, 36°C, d1	15.5	131/136	+17% (vs CTRL)	<0.0001
	sqst-1(ok2892); vha-6p::Q44::yfp		12.4	120/126	-7% (vs CTRL)	0.26
	sqst-1(ok2892); vha-6p::Q44::yfp	1 h, 36°C, d1	13.3	132/135	+8% (vs *sqst-1*- CTRL)	<0.0001
**Muscle PolyQ-expressing animals**						
1	*unc-54p::Q35::yfp*		13.1	88/98		
	unc-54p::Q35::yfp	1 h, 36°C, d1	13.1	90/108	0% (vs CTRL)	0.71
	sqst-1(ok2892); unc-54p::Q35::yfp		12.5	53/90	-4% (vs CTRL)	0.52
	sqst-1(ok2892); unc-54p::Q35::yfp	1 h, 36°C, d1	13.4	58/90	+7% (vs *sqst-1*- CTRL)	0.35
2	*unc-54p::Q35::yfp*		15.1	83/105		
	unc-54p::Q35::yfp	1 h, 36°C, d1	14.3	92/105	-5% (vs CTRL)	0.33
	sqst-1(ok2892); unc-54p::Q35::yfp		14.1	64/105	-7% (vs CTRL)	0.12
	sqst-1(ok2892); unc-54p::Q35::yfp	1 h, 36°C, d1	14.7	74/105	+3% (vs *sqst-1*- CTRL)	0.37

Supplementary Table 2: Lifespan analysis of wild-type (WT, N2) and *sqst-1(ok2892)* animals, and animals expressing tissue-specific PolyQ-stretches (*vha-6* promoter: intestine; *rgef-1* promoter: pan-neuronal; *unc-54* promoter: body-wall muscle) in WT or *sqst-1(ok2892)* mutant background subjected to hormetic heat shock at 36°C (HS) for 1 h on day 1 of adulthood (d1). Animals were fed OP50 bacteria throughout life. Exp: experiment number (in chronological order); MLS: mean lifespan; N: observed deaths/total number of animals; % MLS: percentage change in lifespan compared with control; *P* values by log-rank test. [1]: data depicted in **Fig. 2b** (experiment picked randomly for display).

Supplementary Table 3: Lifespan analyses of *C. elegans* strains overexpressing SQST-1 after hormetic heat shock.

Exp	Strain	Heat shock	MLS (days)	N	% MLS	*P* value
1[1]	WT		14.5	126/132		
	WT	1 h, 36°C, d1	19.2	111/133	+32% (vs WT- CTRL)	<0.0001
	sqIs35[sqst-1p::sqst-1::gfp + unc-122p::rfp] (SQST-1 OE (1))		20.3	116/124	+40% (vs WT- CTRL)	<0.0001
	sqIs35[sqst-1p::sqst-1::gfp + unc-122p::rfp] (SQST-1 OE (1))	1 h, 36°C, d1	20.3	106/116	+0.05% (vs CTRL)	0.4
	sqIs56[rgef-1p::sqst-1::gfp + unc-122p::rfp] (Neuronal SQST-1 OE (1))		16.4	123/127	+13% (vs WT-CTRL)	0.01
	sqIs56[rgef-1p::sqst-1::gfp + unc-122p::rfp] (Neuronal SQST-1 OE (1))	1 h, 36°C, d1	16.6	120/130	+0.9% (vs CTRL)	0.9
2	WT		14.9	112/129		
	WT	1 h, 36°C, d1	17.9	99/128	+20% (vs WT- CTRL)	<0.0001
	sqIs35[sqst-1p::sqst-1::gfp + unc-122p::rfp] (SQST-1 OE (1))		20.6	94/128	+35% (vs WT- CTRL)	<0.0001
	sqIs35[sqst-1p::sqst-1::gfp + unc-122p::rfp] (SQST-1 OE (1))	1 h, 36°C, d1	20.0	102/132	-35% (vs CTRL)	0.4
	sqIs56[rgef-1p::sqst-1::gfp + unc-122p::rfp] (Neuronal SQST-1 OE (1))		17.5	100/141	+18% (vs WT-CTRL)	0.0001
	sqIs56[rgef-1p::sqst-1::gfp + unc-122p::rfp] (Neuronal SQST-1 OE (1))	1 h, 36°C, d1	18.1	84/130	+3% (vs CTRL)	0.5
3	WT		15.4	117/121		
	WT	1 h, 36°C, d1	18.7	101/120	+21% (vs WT- CTRL)	<0.0001
	sqIs36[sqst-1p::sqst-1::gfp + unc-122p::rfp] (SQST-1 OE (2))		17.0	101/120	+10% (vs WT- CTRL)	0.007
	sqIs36[sqst-1p::sqst-1::gfp + unc-122p::rfp] (SQST-1 OE (2))	1 h, 36°C, d1	18.0	114/131	+6% (vs CTRL)	0.3
	sqIs54[rgef-1p::sqst-1::gfp + unc-122p::rfp] (Neuronal SQST-1 OE (2))		16.8	92/120	+9% (vs WT-CTRL)	0.009
	sqIs54[rgef-1p::sqst-1::gfp + unc-122p::rfp] (Neuronal SQST-1 OE (2))	1 h, 36°C, d1	16.2	103/120	-4% (vs CTRL)	0.9

Supplementary Table 3: Lifespan analysis of wild-type (WT, N2) and SQST-1-overexpressing animals subjected to hormetic heat shock at 36°C (HS) for 1 h on day 1 of adulthood (d1). Animals were fed OP50 bacteria throughout life. Exp: experiment number (in chronological order); MLS: mean lifespan; N: observed deaths/total number of animals; % MLS: percentage change in lifespan compared with control; *P* values by log-rank test. [1]: data depicted in **Fig. 3d-e** (experiment picked randomly for display).

Supplementary Table 4: Lifespan analyses of *C. elegans* protein-folding mutants overexpressing SQST-1.

Exp	Strain	MLS (days)	N	% MLS	P value
**Neuronal PolyQ-expressing animals**					
1	*rmIs110[rgef-1p::Q40::yfp]*	15.3	39/100		
	rmIs110[rgef-1p::Q40::yfp]; sqEx147[sqst-1p::sqst-1 + rol-6]	19.0	79/100	+24%	0.0004
	rmIs110[rgef-1p::Q40::yfp]; sqEx146[sqst-1p::sqst-1 + rol-6]	21.7	57/100	+41%	<0.0001
2[1]	*rmIs110[rgef-1p::Q40::yfp]*	11.5	98/119		
	rmIs110[rgef-1p::Q40::yfp]; sqEx147[sqst-1p::sqst-1 + rol-6]	16.5	99/121	+44%	<0.0001
	rmIs110[rgef-1p::Q40::yfp]; sqEx146[sqst-1p::sqst-1 + rol-6]	16.1	104/122	+40%	<0.0001
3	*rmIs110[rgef-1p::Q40::yfp]*	15.0	48/100		
	rmIs110[rgef-1p::Q40::yfp]; sqEx147[sqst-1p::sqst-1 + rol-6]	18.2	81/100	+21%	0.0009
	rmIs110[rgef-1p::Q40::yfp]; sqEx146[sqst-1p::sqst-1 + rol-6]	18.9	68/100	+26%	0.0001
**Neuronal *dyn-1(ts)* mutants**					
1	*dyn-1(ky51) X (ts)*	11.8	140/148		
	dyn-1(ky51) X (ts); sqIs35[sqst-1p::sqst-1::gfp + unc-122p::rfp] (*dyn-1*; SQST-1 OE (1))	13.9	151/151	+18%	0.002
2	*dyn-1(ky51) X (ts)*	14.4	175/178		
	dyn-1(ky51) X (ts); sqIs35[sqst-1p::sqst-1::gfp + unc-122p::rfp] (*dyn-1*; SQST-1 OE (1))	17.4	156/168	+21%	0.0005
3	*dyn-1(ky51) X (ts)*	12.0	152/159		
	dyn-1(ky51) X (ts); sqIs35[sqst-1p::sqst-1::gfp + unc-122p::rfp] (*dyn-1*; SQST-1 OE (1))	18.1	131/153	+51%	0.001
4	*dyn-1(ky51) X (ts)*	10.9	116/119		
	dyn-1(ky51) X (ts); sqIs35[sqst-1p::sqst-1::gfp + unc-122p::rfp] (*dyn-1*; SQST-1 OE (1))	15.3	115/126	+40%	<0.0001
	dyn-1(ky51) X (ts); sqIs36[sqst-1p::sqst-1::gfp + unc-122p::rfp] (*dyn-1*; SQST-1 OE (2))	14.2	112/123	+30%	<0.0001
	dyn-1(ky51) X (ts); sqIs56[rgef-1p::sqst-1::gfp + unc-122p::rfp] (*dyn-1*; neuronal SQST-1 OE (2))	12.4	102/119	+14%	0.03
5	*dyn-1(ky51) X (ts)*	11.1	111/121		
	dyn-1(ky51) X (ts); sqIs35[sqst-1p::sqst-1::gfp + unc-122p::rfp] (*dyn-1*; SQST-1 OE (1))	12.8	114/118	+15%	0.003
	dyn-1(ky51) X (ts); sqIs36[sqst-1p::sqst-1::gfp + unc-122p::rfp] *dyn-1*; SQST-1 OE (2))	13.3	104/120	+20%	0.0004
	dyn-1(ky51) X (ts); sqIs54[rgef-1p::sqst-1::gfp + unc-122p::rfp] (*dyn-1*; neuronal SQST-1 OE (2))	13.0	99/119	+17%	0.003
**Intestinal *let-60(ts)* mutants**					
1	*let-60(ga89) IV (ts)*	13.1	151/155		
	let-60(ga89) IV (ts); sqIs35[sqst-1p::sqst-1::gfp + unc-122p::rfp] (*let-60*; SQST-1 OE (1))	15.0	159/161	+15%	0.01
2	*let-60(ga89) IV (ts)*	11.6	161/165		
	let-60(ga89) IV (ts); sqIs35[sqst-1p::sqst-1::gfp + unc-122p::rfp] (*let-60*; SQST-1 OE (1))	17.3	136/149	+49%	<0.0001
**Muscle *unc-54(ts)* and *unc-15(ts)* mutants**					
1	*unc-54(e1301) I (ts)*	7.1	167/168		
	unc-54(e1301) I (ts); sqIs35[sqst-1p::sqst-1::gfp + unc-122p::rfp] (*unc-54*; SQST-1 OE (1))	6.9	35/40	-3%	0.9
2	*unc-54(e1301) I (ts)*	12.3	167/170		
	unc-54(e1301) I (ts); sqIs35[sqst-1p::sqst-1::gfp + unc-122p::rfp] (*unc-54*; SQST-1 OE (1))	14.0	128/135	+14%	0.006
3	*unc-15(e1402) I (ts)*	13.9	135/152		
	unc-15(e1402) I (ts)); sqIs35[sqst-1p::sqst-1::gfp + unc-122p::rfp] (*unc-15*; SQST-1 OE (1))	13.1	128/138	-6%	0.8

Supplementary Table 4: Lifespan analysis of protein-folding mutants overexpressing SQST-1. Animals either express neuronal PolyQ (*rgef-1* promoter: pan-neuronal) or carry temperature-sensitive (ts) missense mutations in neuronal GTPase dynamin (*dyn-1(ts)*), intestinal Ras (*let-60(ts)*), body-wall myosin (*unc-54(ts)*) and body-wall paramyosin (*unc-15(ts)*). Animals were fed OP50 bacteria throughout life. Exp: experiment number (in chronological order); MLS: mean lifespan; N: observed deaths/total number of animals; % MLS: percentage change in lifespan compared with control; *P* values by log-rank test. [1]: data depicted in **Fig. 3h** (experiment picked randomly for display).

Supplementary Table 5: Autophagic vesicle diameter in wild-type and SQST-1-overexpressing animals.

Tissue	WT				SQST-1 OE				*P* value
	Min	Median	Max	N	Min	Median	Max	N	
Nerve-ring neurons[1]	260 nm	**720 nm**	1840 nm	519	390 nm	**1120 nm**	3160 nm	1196	<0.0001
Body-wall muscle	260 nm	**400 nm**	600 nm	28	-	**-**	-	-	
Proximal intestinal cells[2]	200 nm	**470 nm**	1200 nm	133	270 nm	**670 nm**	2200 nm	174	<0.0001
Terminal pharyngeal bulb[3]	260 nm	**500 nm**	1200 nm	81	130 nm	**730 nm**	1730 nm	161	<0.0001

Supplementary Table 5: The diameter of GFP::LGG-1/Atg8-positive punctae was measured in wild-type (WT, N2) animals and animals overexpressing SQST-1 (SQST-1 OE) (*sqIs13[lgg-1p::gfp::lgg-1 + odr-1p::rfp]; sqEx146[sqst-1p::sqst-1 + rol-6]* and *sqIs24[rgef-1p::gfp::lgg-1 + unc-122p::rfp]; sqEx147[sqst-1p::sqst-1 + rol-6]* for neurons). The body-wall muscle was difficult to see in SQST-1-overexpressing animals and could not be analyzed. Min: smallest diameter; Median: Median diameter; Max: largest diameter; N: number of GFP::LGG-1/Atg8-positive punctae, combined from n=2-6. *P* values by Wilcoxon Signed rank test after autophagic vesicles were binned according to diameter. [1]: data depicted in **Fig. 4e**, [2]: data depicted in **Supplementary Fig 12f**, [3]: data depicted in **Supplementary Fig 12g**.

Supplementary Table 6: Sequences of quantitative RT-PCR primers used in this study.

Gene	Primer sequence 5′ → 3′	
**Experimental genes**		
atg-18	Fwd	AAA TGG ACA TCG GCT CTT TG
	Rev	TGA TAG CAT CGA ACC ATC CA
bec-1	Fwd	TCGAGCTCCCACTCTTTGGCG
	Rev	TGA CAC CAT TGT CAA CCA GTG
lgg-1	Fwd	ACC CAG ACC GTA TTC CAG TG
	Rev	ACG AAG TTG GAT GCG TTT TC
lmp-1	Fwd	ATCCGCCACCGCTTCGCATT
	Rev	TCGAGCTCCCACTCTTTGGCG
vha-15	Fwd	CGA GGT TCG TTC CGG ACG TCT T
	Rev	CCT CGG CAG TCA GGA GAC GC
vha-16	Fwd	AGG CGC TGA CTC GCG GAC TT
	Rev	TGG TCT CTG GTG AAG AGT TCC GGT G
vps-34	Fwd	GGA TTG TAT TCG TGA AGC AAT G
	Rev	GTC AAG ATG ACG ATC ACC AAG
hsp-70 (C12C8.1)	Fwd	ACT CAT GTG TCG GTA TTT ATC
	Rev	ACG GGC TTT CCT TGT TTT
sqst-1	Fwd	CAG CCC TAC CAT CAG TGA GC
	Rev	CTG AAC TTC CTC GAC AAC AGG
**Housekeeping genes used for normalization**		
ama-1	Fwd	TGG AAC TCT GGA GTC ACA CC
	Rev	CAT CCT CCT TCA TTG AAC GG
pmp-3	Fwd	GTTCCCGTGTTCATCACTCAT
	Rev	ACACCGTCGAGAAGCTGTAGA
cdc-42	Fwd	CTGCTGGACAGGAAGATTACG
	Rev	CTCGGACATTCTCGAATGAAG
nhr-23	Fwd	CAG AAA CAC TGA AGA ACG CG
	Rev	CGA TCT GCA GTG AAT AGC TC

3. Supplementary Methods

Western Blot

Total protein was extracted from the indicated number of worms (10-200 animals were handpicked or worms for >200 worms were washed off multiple plates) on Day 1 of adulthood grown on OP50 bacteria at 20°C. Animals were washed thoroughly in M9 buffer until OP50 bacteria were completely removed. Proteins were extracted by lysing animals by up to 10 cycles of freeze-thaw in Laemmli buffer containing 5% β-mercaptoethanol, and 10-20 min of boiling at 95°C. The extract was loaded onto 4-12% Bis-Tris protein gel (Thermo Fisher Scientific) for separation and transferred to a PVDF membrane (Millipore). For anti-GFP, anti-SQST-1, and anti-PolyQ blots, membranes were blocked with 5% milk in TBS-T and immunoblotting was performed using primary mouse anti-GFP (diluted 1:1000; Santa Cruz Biotechnology SC-9996), rabbit anti-SQST-1[7] (diluted 1:3000) or mouse anti-PolyQ (diluted 1:1000, Sigma-Aldrich P1874). For anti-ubiquitin blots, membranes were blocked with 5% BSA and immunoblotting was performed using horseradish peroxidase-conjugated mouse monoclonal antibody against mono-and poly-ubiquitinated conjugates (diluted 1:1000, Enzo Life Sciences FK2). The blots were developed using enhanced chemiluminescent reagent (Thermo Fisher Scientific). To normalize for loading, the blots were stripped for 1 h using stripping buffer (1.5% glycine, pH 2.5) and probed with primary goat anti-tubulin antibody (diluted 1:1000; Santa Cruz Biotechnology SC-12836) or rabbit anti-actin antibody (diluted 1:1000; abcam ab8227). Secondary horseradish peroxidase-conjugated antibodies were donkey anti-goat antibody (diluted 1:10,000; Santa Cruz Biotechnology SC-2020), goat anti-rabbit antibody (diluted 1:10,000; Cell Signaling 7074S), or rabbit anti-mouse (diluted 1:10,000, Cell Signaling 7076S). Western Blots were quantified using Image Lab, version 6 by Bio-Rad Laboratories, Inc.

Quantitative reverse transcriptase PCR

Quantitative reverse transcriptase (RT) PCR was performed as previously described[8]. Briefly, total RNA was isolated from a synchronized population of ~2,000 one-day-old nematodes raised on OP50 bacteria and maintained under control conditions or subjected to heat shock for 1 h at 36°C.

For quantitative PCR analyses of older animals, the synchronized animals were washed off daily with M9 medium, adult animals were sedimented by gravity and the floating larvae were aspirated. This washing step was repeated until no more floating larvae were detected. The adult animals were re-seeded onto 10 cm NGM plates with OP50 bacteria or harvested on the desired day of adulthood. After harvesting, the animals were flash frozen in liquid nitrogen. RNA was extracted with TRIzol (Life Technologies), purified using a Qiagen RNeasy kit, and subjected to an additional DNA digestion step (Qiagen DNase I kit). Reverse transcription (1 μg RNA per sample) was performed using M-MuLV reverse transcriptase and random 9-mer primers (New England Biolabs)[9].

Quantitative RT-PCR was performed using SYBR Green Master Mix in an LC480 LightCycler (Roche). A standard curve was obtained for each primer set by serially diluting a mixture of different complementary DNAs and the standard curves were used to convert the observed CT values to relative values. Three biological samples were analyzed, each with three technical replicates. mRNA levels of target genes were normalized to

the mean of the following housekeeping genes: *ama-1* (large subunit of RNA polymerase II), *nhr-23* (nuclear hormone receptor), *cdc-42* (Rho-GTPase) and *pmp-3* (putative ABC transporter)[9,10]. Primer sequences are listed in **Supplementary Table 6**. Data are displayed as relative values compared with controls. Data were analyzed using multiple t-test or one-way ANOVA (GraphPad Prism).

Healthspan parameters

Thrashing ability (i.e., swimming), pharyngeal pumping, progeny production, and body length were assayed as measures of healthspan. For swimming assays, animals on the indicated days of adulthood were transferred onto an NGM media plate containing a drop of M9 medium, and body bends of 14-20 animals were counted for 20 s on a Leica stereoscope. Pharyngeal pumping was measured on the indicated days of adulthood by counting the grinder movements in the terminal pharyngeal bulb of 14–20 animals for 15 s on a Leica stereoscope. For the assessment of a progeny production profile, 10 animals were singled on 6 cm NGM plates at the L4 larval stage and transferred daily onto fresh plates during the self-fertile reproductive span. The number of eggs/larvae produced by each animal per day was counted. For measurements of body length, 10-12 animals on day 1 of adulthood were anaesthetized with M9 medium containing 0.1% NaN_3 and aligned for imaging with a Leica DFC310 FX camera. Image analysis was performed with ImageJ software (National Institutes of Health) by tracing the length of the individual animals. Data were analyzed by student's *t*-test or one-way ANOVA as applicable (GraphPad Prism).

Differential Detergent Extraction

Animals from 5 10 cm NGM plates were bleached and left overnight in M9 buffer at 20°C for hatching. The next day, L1 larvae were seeded on 10 cm semi-High Growth Media (HGM) plates (1.2% Peptone, 51mM NaCL, 25mM [PO4], 5ug/ml Cholesterol, 1mM $CaCl_2$, 1mM $MgCl_2$, 2.5% Agar) coated with OP50 and kept at 20°C until day 1 of adulthood. On day 1 of adulthood animals were either subjected to a 1h heat shock at 36°C or kept at control conditions. Animals were washed off and reseeded on 10 cm semi-High Growth Media (HGM) plates coated with OP50 bacteria and containing 100 μg/ml 5-Fluoro-2′-deoxyuridine (FuDR) (Acros Organics) to prevent progeny development. On day 7 of adulthood animals were collected with M9 buffer. 100 μl of worm pellet was mixed with 100 μl of 0.5 mm Zirconia beads and 200 μl of lysis buffer (10 mM Tris-HCl, pH 8.0, 150 mM NaCl, 0.1% SDS, Complete Proteinase Inhibitor with 2 mM EDTA, 0.5 mM PMSF) was added. Lysates were generated by homogenization using a Precellys Evolution homogenizer (Bertin Technologies) (3x 6000 rpm, 45 s, with 30s pause). Lysates were incubated at room temperature for 1 h. Protein concentration of lysates was determined using Pierce 660 nm Quantification assay. 200 μg of protein lysate was placed into 200 μl Beckman Polycarbonate Centrifuge Tube and subjected to ultracentrifugation (Beckman TL-10, 100,000 g, 20 min, 4°C) using Beckman rotor TLA100. The supernatant was transferred to a fresh tube and kept for Western Blot analysis. The pellet was resuspended in 100 μl lysis buffer with increasing SDS concentration (0.25%, 0.5%, 0.75%, 1.0%, 2% SDS), subjected to heating and cooling (15 min 95°C, 15 min 25°), and spun down again using the TL-10 ultracentrifuge. After the last centrifugation step, the pellet was dissolved in 100 μl urea buffer (30 mM

Tris-HCl, pH 8.0, 150 mM NaCl, 2% SDS, 1% Triton X-100, 7 M Urea, 2 M Thiourea, 0.5 mM PMSF, Complete Proteinase Inhibitor (Roche)). 8 μl of the collected fractions were mixed with 3 μl of 4x sample buffer (250 mM Tris/HCl, pH 6.8, 8% (w/v) SDS, 40% (v/v) glycerol, 0.02% (w/v) Bromophenol Blue and 4% (v/v) 2-mercaptoethanol), boiled for 5 min and loaded onto 4-12% Bis-Tris gels and subjected to Western Blot analysis. Western Blots were quantified using Image Lab, version 6 by Bio-Rad Laboratories, Inc. Briefly, PolyQ protein intensity from all samples was set to 100% and the fraction of soluble and insoluble PolyQ was calculated. Data were analyzed by two-way ANOVA (GraphPad Prism).

Proteasome activity

Animals were bleached and left overnight in M9 buffer at 20°C for hatching. The next day, L1 larvae were seeded on 10 cm NGM plates coated with OP50 bacteria and kept at 20°C until day 1 of adulthood. *glp-1(e2144)* animals were kept at 25°C for development. On day 1 of adulthood animals were washed off and reseeded on plates containing 100 μg/ml 5-Fluoro-2′-deoxyuridine (FuDR) (Acros Organics) to prevent progeny development. On day 3 of adulthood animals were collected with M9 buffer, and the worm pellet was resuspended in proteasome buffer (50 mM Tris-HCl, pH 7.5, 10% glycerol, 5 mM MgCl2, 0.5 mM EDTA, 2 mM ATP, and 1 mM dithiothreitol). Lysates were generated by glass bead disruption on ice using a Precellys 24 homogenizer (Bertin Technologies). Debris were removed by two centrifugation steps, the first at 6000g for 5 min, the second at 13000g spin for 15 min, both at 4°C. Protein concentration was measured and a total of 20 µg of total protein lysate was transferred to a 96-well microtiter plate (BD Falcon) and incubated with fluorogenic Suc-LLVY-AMC substrate (Enzo, BML-P802) for the chymotrypsin activity of the 20S proteasome. Fluorescence (370 nm excitation, 445 nm emission) was monitored on a microplate fluorometer (Perkin Elmer EnSpire) every 5 min for 1 h at 25°C. The slope for the fluorescence increase is calculated by linear regression using Microsoft Excel.

Paralysis

Paralysis was assessed in temperature-sensitive aggregation mutants MAH477 (*dyn-1(ky51)*), MAH482 (*dyn-1(ky51); sqls35[sqst-1p::sqst-1::gfp+unc-122p::rfp (1)*), MAH786 (*dyn-1(ky51); sqst-1p::sqst-1::gfp+unc-122p::rfp (2)*), MAH838 *(dyn-1(ky51); rgef-1p::sqst-1::gfp+unc-122p::rfp (2)*), MAH478 (*let-60(ga89)*), MAH488 (*let-60(ga89); sqst-1p::sqst-1::gfp+unc-122p::rfp (1)*), CB1301 (*unc-54(e1301)*), and MAH460 (*unc-54(e1301); sqst-1p::sqst-1::gfp+unc-122p::rfp (1)).* Animals were raised and kept at 15°C for analyses at the permissive temperature. For analyses at the restrictive temperature, animals were raised at 20°C until day 0 of adulthood. At this time, 10-30 animals of each strain were placed on three-four 6 cm NGM plates and were incubated at 25°C until day 3 of adulthood. Paralysis was scored when animals did not move voluntarily.

4. Supplementary References

1 Manil-Segalen, M. *et al.* The C. elegans LC3 acts downstream of GABARAP to degrade autophagosomes by interacting with the HOPS subunit VPS39. *Developmental cell* **28**, 43-55, doi:10.1016/j.devcel.2013.11.022 (2014).

2 Kuma, A., Matsui, M. & Mizushima, N. LC3, an autophagosome marker, can be incorporated into protein aggregates independent of autophagy: caution in the interpretation of LC3 localization. *Autophagy* **3**, 323-328 (2007).

3 Chang, J. T., Kumsta, C., Hellman, A. B., Adams, L. M. & Hansen, M. Spatiotemporal regulation of autophagy during Caenorhabditis elegans aging. *Elife* **6**, doi:10.7554/eLife.18459 (2017).

4 Lin, L. *et al.* The scaffold protein EPG-7 links cargo-receptor complexes with the autophagic assembly machinery. *J Cell Biol* **201**, 113-129, doi:10.1083/jcb.201209098 (2013).

5 Kamath, R. S., Martinez-Campos, M., Zipperlen, P., Fraser, A. G. & Ahringer, J. Effectiveness of specific RNA-mediated interference through ingested double-stranded RNA in Caenorhabditis elegans. *Genome Biol* **2**, RESEARCH0002, doi:10.1186/gb-2000-2-1-research0002 (2001).

6 Vilchez, D. *et al.* RPN-6 determines C. elegans longevity under proteotoxic stress conditions. *Nature* **489**, 263-268, doi:10.1038/nature11315 (2012).

7 Springhorn, A. & Hoppe, T. Western blot analysis of the autophagosomal membrane protein LGG-1/LC3 in Caenorhabditis elegans. *Methods in enzymology* **619**, 319-336, doi:10.1016/bs.mie.2018.12.034 (2019).

8 Kumsta, C., Chang, J. T., Schmalz, J. & Hansen, M. Hormetic heat stress and HSF-1 induce autophagy to improve survival and proteostasis in C. elegans. *Nat Commun* **8**, 14337, doi:10.1038/ncomms14337 (2017).

9 Taubert, S., Van Gilst, M. R., Hansen, M. & Yamamoto, K. R. A Mediator subunit, MDT-15, integrates regulation of fatty acid metabolism by NHR-49-dependent and -independent pathways in C. elegans. *Genes Dev* **20**, 1137-1149, doi:20/9/1137 [pii]10.1101/gad.1395406 (2006).

10 Lapierre, L. R. *et al.* The TFEB orthologue HLH-30 regulates autophagy and modulates longevity in Caenorhabditis elegans. *Nat Commun* **4**, 2267, doi:10.1038/ncomms3267 (2013).
