# Supplementary material for: The autophagy receptor p62/SQST-1 promotes proteostasis and longevity in C. elegans by inducing autophagy
Source: Nat Commun. 2019 Dec 11;10:5648. doi: 10.1038/s41467-019-13540-4 (PMC6906454; doi:10.1038/s41467-019-13540-4)
Supplement: Supplementary file 3 — Description of Additional Supplementary Files [file 41467_2019_13540_MOESM3_ESM.pdf]

Description of Additional Supplementary Files

File Name: Supplementary Data 1
Description: Lifespan analyses of sqst-1 mutants in combination with lifespan-extending genetic regimens.

File Name: Supplementary Data 2
Description: Lifespan analyses of C. elegans strains overexpressing SQST-1.

File Name: Supplementary Data 3
Description: Lifespan analyses of C. elegans strains overexpressing SQST-1 subjected to autophagy gene RNAi.

File Name: Supplementary Data 4
Description: C. elegans strains used in this study.
