# Supplementary material for: The autophagy receptor p62/SQST-1 promotes proteostasis and longevity in C. elegans by inducing autophagy
Source: Nat Commun. 2019 Dec 11;10:5648. doi: 10.1038/s41467-019-13540-4 (PMC6906454; doi:10.1038/s41467-019-13540-4)
Supplement: Supplementary file 8 — Reporting Summary [file 41467_2019_13540_MOESM8_ESM.pdf]

nature research

Corresponding author(s): Malene Hansen, Caroline Kumsta

Last updated by author(s): Nov 5, 2019

Reporting Summary

Nature Research wishes to improve the reproducibility of the work that we publish. This form provides structure for consistency and transparency in reporting. For further information on Nature Research policies, see Authors & Referees and the Editorial Policy Checklist.

Statistics

For all statistical analyses, confirm that the following items are present in the figure legend, table legend, main text, or Methods section.

n/a	Confirmed	
☐	☒	The exact sample size (*n*) for each experimental group/condition, given as a discrete number and unit of measurement
☒	☐	A statement on whether measurements were taken from distinct samples or whether the same sample was measured repeatedly
☐	☒	The statistical test(s) used AND whether they are one- or two-sided<br>*Only common tests should be described solely by name; describe more complex techniques in the Methods section.*
☒	☐	A description of all covariates tested
☐	☒	A description of any assumptions or corrections, such as tests of normality and adjustment for multiple comparisons
☐	☒	A full description of the statistical parameters including central tendency (e.g. means) or other basic estimates (e.g. regression coefficient) AND variation (e.g. standard deviation) or associated estimates of uncertainty (e.g. confidence intervals)
☒	☐	For null hypothesis testing, the test statistic (e.g. *F*, *t*, *r*) with confidence intervals, effect sizes, degrees of freedom and *P* value noted<br>*Give P values as exact values whenever suitable.*
☒	☐	For Bayesian analysis, information on the choice of priors and Markov chain Monte Carlo settings
☒	☐	For hierarchical and complex designs, identification of the appropriate level for tests and full reporting of outcomes
☒	☐	Estimates of effect sizes (e.g. Cohen's *d*, Pearson's *r*), indicating how they were calculated

Our web collection on statistics for biologists contains articles on many of the points above.

Software and code

Policy information about availability of computer code

Data collection	Zen 2.3 was used to acquire images.
Data analysis	Image J was used for image processing. Statistical analysis was conducted using Graph Pad, Stata and Oasis 2c (Han et al (2016) Oncotarget 7:56147-56152).

For manuscripts utilizing custom algorithms or software that are central to the research but not yet described in published literature, software must be made available to editors/reviewers. We strongly encourage code deposition in a community repository (e.g. GitHub). See the Nature Research guidelines for submitting code & software for further information.

Data

Policy information about availability of data

All manuscripts must include a data availability statement. This statement should provide the following information, where applicable:

- Accession codes, unique identifiers, or web links for publicly available datasets
- A list of figures that have associated raw data
- A description of any restrictions on data availability

There are no accession codes, unique identifiers or weblinks in our study and no restrictions on data availability. All Data figures and panels have raw data associated with them that is provided as an excel "Source file", expect representative images.

Field-specific reporting

Please select the one below that is the best fit for your research. If you are not sure, read the appropriate sections before making your selection.

☒ Life sciences ☐ Behavioural & social sciences ☐ Ecological, evolutionary & environmental sciences

For a reference copy of the document with all sections, see nature.com/documents/nr-reporting-summary-flat.pdf

Life sciences study design

All studies must disclose on these points even when the disclosure is negative.

Sample size	Sample size was not predetermined using any statistical method.
Data exclusions	No data exclusions occurred.
Replication	Multiple trials/biological of each experiment were conducted. Replicates are indicated in the figure legends or where combined for data representation.
Randomization	Animals were randomly chosen for analysis.
Blinding	Most experiments were conducted by multiple investigators to confirm results. Animals used for foci and aggregate counts were selected in a blinded manner.

Reporting for specific materials, systems and methods

We require information from authors about some types of materials, experimental systems and methods used in many studies. Here, indicate whether each material, system or method listed is relevant to your study. If you are not sure if a list item applies to your research, read the appropriate section before selecting a response.

Materials & experimental systems

n/a	Involved in the study	
☐	☒	Antibodies
☒	☐	Eukaryotic cell lines
☒	☐	Palaeontology
☐	☒	Animals and other organisms
☒	☐	Human research participants
☒	☐	Clinical data

Methods

n/a	Involved in the study	
☒	☐	ChIP-seq
☒	☐	Flow cytometry
☒	☐	MRI-based neuroimaging

Antibodies

Antibodies used	1. Anti-GFP (diluted 1:1000; Santa Cruz Biotechnology, SC-9996),<br>2. Anti-SQST-1 (diluted 1:3000, Reference: Springhorn et al, Methods in Enzymology, 2019),<br>3. Anti mono-and poly-ubiquitinated conjugates (diluted 1:1000 FK2; Enzo Life Sciences),<br>4. Anti Monoclonal Anti-Polyglutamines (diluted 1:1000, Sigma-Aldrich, P1874)
Validation	1. Anti-GFP has been vastly used and validated, including in C. elegans. In 2019 alone, more than 30 publications have used this antibodies for GFP detection in transgenic C. elegans, expressing GFP.<br>2. Anti-SQST-1 has been validated in Reference: Springhorn et al, Methods in Enzymology, 2019.<br>3. Anti-mono-and poly-ubiquitinated conjugates, is species independent, detects K29-, K48-, and K63-linked mono- and polyubiquitinylated proteins, most cited multi-ubiquitin antibody with over 500 citations, validated for WB, IP, IHC, and ELISA applications.<br>4. Anti-Polyglutamine has been used to detect PolyQ stretches expressed in C. elegans (Reference Imanikia et al., Current Biology, 2019) and has been recommended by the reviewers. See peer-review file for antibody validation.

Animals and other organisms

Policy information about studies involving animals; ARRIVE guidelines recommended for reporting animal research

Laboratory animals	C. elegans. The strains used are listed in Supplementary Table 10.
Wild animals	n/a

Field-collected samples	n/a
Ethics oversight	n/a

Note that full information on the approval of the study protocol must also be provided in the manuscript.
